# Global mining has undermined forest conservation within and beyond protected areas

**DOI:** 10.1038/s41467-026-74859-3

**Published:** 2026-06-24

**Authors:** He Ren, Yihua Hu, Tingting He, Yanling Zhao, Youpeng Lu, Fashuai Li, Maoxin Zhang, Jianyong Zhang, Hongtao Cao, Huicai Yang, Jin Wu, Zhipei Sun, Jianyu Wang, Yuwei Chen

**Affiliations:** 1 Academy of Eco-Civilization Development for Jing-Jin-Ji Megalopolis, Tianjin Normal University, Tianjin, PR China; 2 State Key Laboratory of Pulsed Power Laser Technology, Hefei, PR China; 3 Advanced Laser Technology Laboratory of Anhui Province, Hefei, PR China; 4 Department of Land Management, Zhejiang University, Hangzhou, PR China; 5 Institute of Land Reclamation and Ecological Restoration, China University of Mining and Technology (Beijing), Beijing, PR China; 6 Advanced Interdisciplinary Institute of Satellite Applications, Beijing Normal University, Beijing, PR China; 7 Department of Electronics and Nanoengineering and QTF Centre of Excellence, Aalto University, Aalto, Finland; 8 Hangzhou Institute of Advanced Studies, University of Chinese Academy of Science, Hangzhou, PR China

**Keywords:** Forestry, Forestry

## Abstract

Protected areas are widely regarded as a cornerstone of global forest conservation, yet growing mineral demand intensifies mining pressure on them. Here, we present a global assessment of mining-induced forest loss within and beyond protected areas from 2001 to 2020. We find that approximately 11% (~1372 km^2^) of global mining-induced forest loss occurs within protected areas, with this proportion increasing to ~19% during 2016–2020. Furthermore, ~27% (544) of mines associated with forest loss within protected areas remain active throughout the study period. Mining impacts also extend beyond protected area boundaries, where forest loss in the surrounding 10-km buffer zones (~4113 km^2^) is nearly three times that observed within protected areas. Temporal analyses further reveal that mining increasingly affects forests with higher biomass density over time. These findings demonstrate growing mining pressure on protected forests and highlight the need to mitigate impacts both within and beyond protected area boundaries.

## Abstract (web summary)

Protected areas help conserve forests but are increasingly threatened by mining. This study suggests that mining-induced forest loss is expanding both within and beyond protected areas and is increasingly affecting high-biomass forests.

## Introduction

Protected areas (PAs) are crucial for biodiversity conservation^1,2^. To enhance biodiversity protection, reduce habitat loss, and mitigate human encroachment, the United Nations has set ambitious targets. For instance, the recent Kunming-Montreal Global Biodiversity Framework (GBF) aims for the “30 × 30” target, seeking to conserve 30% of the world’s terrestrial and marine areas by 2030. PAs also play a pivotal role in global efforts to reduce deforestation, contributing to curbing forest cover loss and increasing forest carbon stocks^3–6^. Research indicates that each 1% expansion of a country’s protected or biodiversity conservation areas can increase its total forest cover by 0.03%^7^. Although widely recognized as effective conservation tools, PAs face significant pressures from human industrial activities^8^. A recent study reveals that forest loss within global PA boundaries has accelerated, with PAs contributing to ~13.57% of global forest loss between 2001 and 2020^9^.

Among these pressures, mining has emerged as a particularly urgent driver of forest loss. While agriculture, forestry, and livestock production remain the dominant drivers of global deforestation^10–13^, mining, although accounting for a relatively small share of forest loss, frequently overlaps with biodiversity hotspots and protected areas^14–20^. Despite lacking mining permits and restoration plans, mining-induced forest loss within PAs continues to occur^21–23^. Compared to other land-use changes, mining typically results in the complete removal of forest components, including the mineral soil, and may therefore pose a disproportionate impact on species diversity and ecosystem stability, given the ecological importance of these critical regions. In addition, mining exerts indirect pressures that often extend far beyond its immediate footprint, including infrastructure development, road expansion, and the expansion of other land uses^24–26^, with impacts reaching distances of up to 70 km^27^.

~14% of PAs contain mine sites within or near their boundaries^16^, and since 2000, nearly half of global mineral extraction has occurred within 20 km of PAs^17^. A recent study further indicates that more than 7000 PAs worldwide have been or are currently affected by mining activities^28^. These findings provide global evidence of the growing exposure of PAs to mining pressure. However, most existing assessments infer potential impacts based primarily on spatial overlap between mining activities and PAs, which does not capture the actual extent or dynamics of mining-induced forest loss. Although an increasing number of regional case studies have documented forest loss within and beyond PAs attributable to mining^29–33^, these studies remain spatially limited and lack a consistent global assessment. This limitation is particularly critical for areas surrounding PAs, where mining pressures may be closely linked to those inside PA boundaries and disproportionately affect adjacent habitats and ecosystems that are often more vulnerable than the PAs themselves^34,35^. As global demand for critical minerals continues to surge under the ongoing energy transition^36–40^, these pressures are expected to intensify further, amplifying risks to forest ecosystems associated with PAs. Despite these growing concerns, a systematic and spatially explicit global quantification of mining-induced forest loss both within and beyond PAs remains lacking. Addressing this gap is essential for accurately evaluating the effectiveness of PAs under increasing resource extraction pressure and for informing evidence-based conservation strategies.

Here, we provide a systematic global assessment of mining-induced forest loss both within and beyond PAs. By integrating the Global Forest Change (GFC) dataset^41^, global mining land-use datasets^42,43^, and PA boundaries, we quantify the spatiotemporal patterns of mining-induced forest loss from 2001 to 2020. In estimating forest loss within PAs, we account for the year of PA establishment, ensuring that forest loss is attributed only to the period after each PA was designated. We further delineate buffer zones outside PA boundaries at 1-km intervals, ranging from 1 to 10 km, to characterize forest loss beyond PAs. We quantify the number of mine sites contributing to forest loss within PAs and compare patterns across IUCN management categories. We also develop an approach based on forest loss dynamics to identify active mine sites that exert persistent pressure on forests within PAs, and integrate a global biomass density map^44^ to examine temporal trends in biomass density associated with mining-induced forest loss. Our results reveal that mining threatens forest conservation both within and beyond PAs, and highlight the importance of formulating more effective management strategies for PAs from a global perspective.

## Results

### Global mining-induced forest loss

**Fig. 1:**
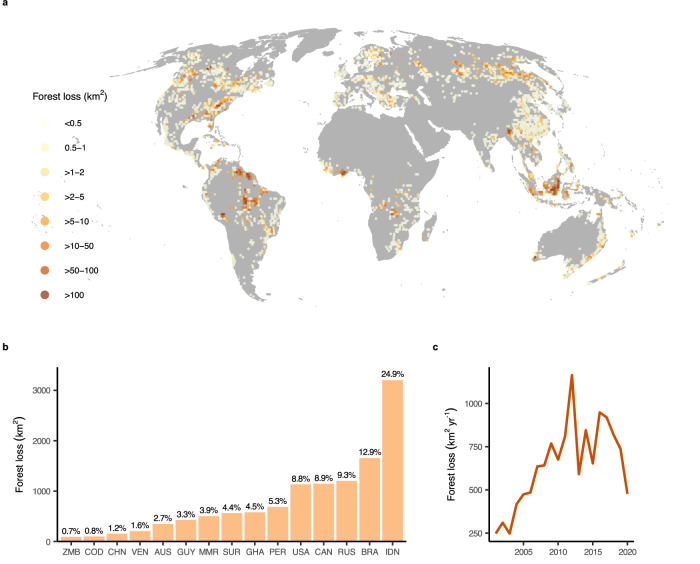
Mining-induced forest loss. **a** Cumulative mining-induced forest loss from 2001 to 2020, aggregated to 1° grid cells for visualization. **b** The fifteen countries with the highest cumulative mining-induced forest loss from 2001 to 2020. Labels above bars indicate each country’s contribution to the global total. IDN, Indonesia; BRA, Brazil; RUS, Russia; CAN, Canada; USA, United States; PER, Peru; GHA, Ghana; SUR, Suriname; MMR, Myanmar; GUY, Guyana; AUS, Australia; VEN, Venezuela; CHN, China; COD, Democratic Republic of the Congo; ZMB, Zambia. **c** Annual mining-induced forest loss from 2001 to 2020. The underlying world map is sourced from https://www.naturalearthdata.com/ and plotted using medium-scale country boundary data from the R package rnaturalearth (v. 1.0.1)^79^. Source data are provided as a Source Data file.

Mining caused a direct forest loss of ~12,865 km^2^ from 2001 to 2020, with an average annual loss of ~643 km^2^ (Fig. 1a). Tropical regions, particularly Kalimantan, the northeastern Amazon region, central Ghana, and the Democratic Republic of the Congo (DRC), were identified as hotspots of forest loss. Significant forest loss was also observed in eastern North America, western Canada, Siberia, and the Indochina Peninsula. Indonesia and Brazil accounted for over one-third of global mining-induced forest loss, with Indonesia experiencing the most substantial losses (~3202 km^2^) from 2001 to 2020 (Fig. 1b). Mining-intensive nations such as the United States, Russia, Canada, China, and Australia also experienced varying degrees of forest loss. Forest loss exhibited a significant increasing trend from 2001 to 2012 (MK test, Z = 4.05, p < 0.001, Sen’s slope=63.8 km^2 ^yr^−1^, 95% CI [54.5, 83.4]; Fig. 1c). However, the rate of forest loss slowed after 2012, primarily due to decreased loss in both Asia and South America (Supplementary Fig. 1). Although mining-induced forest loss in South America declined after 2017, it still exceeded 200 km^2^ in 2020, representing nearly four times the amount recorded in 2001. Forest loss in this region accounted for ~40% of the global total in 2020, underscoring the significant threat posed by mining in South America.

### Mining-induced forest loss within protected areas

**Fig. 2:**
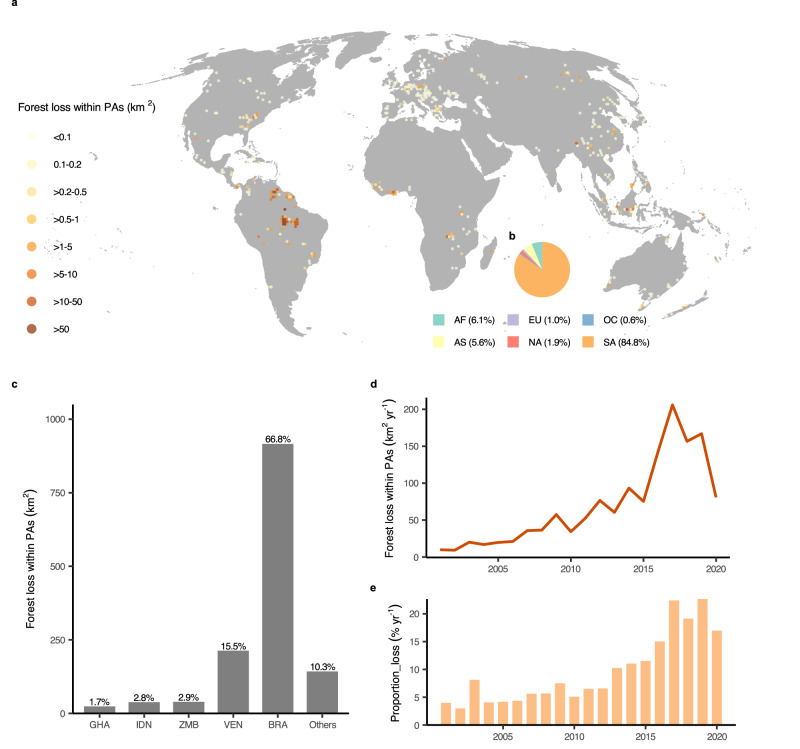
Mining-induced forest loss within protected areas (PAs). **a** Cumulative mining-induced forest loss within PA boundaries from 2001 to 2020, aggregated to 1° grid cells for visualization. **b** Continental contributions to cumulative mining-induced forest loss within PAs. AF, Africa; AS, Asia; EU, Europe; NA, North America; OC, Oceania; SA, South America. **c** The five countries with the highest cumulative mining-induced forest loss within PAs. Percentages indicate each country’s contribution to the global total. BRA, Brazil; VEN, Venezuela; ZMB, Zambia; IDN, Indonesia; GHA, Ghana. “Others” represents all remaining countries. **d** Annual mining-induced forest loss within PAs from 2001 to 2020. **e** Annual Proportion_loss from 2001 to 2020. Proportion_loss is defined as the proportion of global mining-induced forest loss occurring within PAs (Eq.1 in Methods). The underlying world map is sourced from https://www.naturalearthdata.com/ and plotted using the medium-scale country boundary data from the R package rnaturalearth (v. 1.0.1)^79^. Source data are provided as a Source Data file.

We found that ~11% (~1372 km^2^) of mining-induced forest loss occurred within PA boundaries (Fig. 2a). The majority of this loss was concentrated in South America (~84.8%; Fig. 2b), followed by Africa (~6.1%) and Asia (~5.6%). At the national level, Brazil, Venezuela, Zambia, Indonesia, and Ghana were the primary contributors, accounting for ~89% of the forest loss within PAs, with all other countries contributing less than 1% each (Fig. 2c). Although the annual rate of forest loss decreased after 2012, deforestation pressure within PAs continued to intensify until 2017 (MK test, Z = 4.73, *p* < 0.001, Sen’s slope=6.3 km^2 ^yr^−1^, 95% CI [4.7, 9.4]; Fig. 2d) and was most pronounced in Africa and South America (Supplementary Fig. 2). More importantly, we found that the proportion of mining-induced forest loss occurring within PAs (Proportion_loss) increased significantly over the study period (MK test, Z = 4.89, p < 0.001, Sen’s slope=0.80% yr^−1^, 95% CI [0.53, 1.15]; Fig. 2e), reaching ~19% in the most recent five years (2016–2020). This increase was primarily attributable to the intensification of forest loss in South America during 2010–2020 (MK test, Z = 3.42, p < 0.001, Sen’s slope=3.1% yr^−1^, 95% CI [1.5, 3.8]; Supplementary Fig. 3). Despite the increasing extent of forest loss, forest recovery within PAs remained extremely limited. Using forest gain information from the GFC dataset, we assessed post-mining forest recovery as described in Supplementary Note 1. We found that forest gain within PAs accounted for only 2.45% of the total forest loss during 2001–2012 (Supplementary Table 1).

**Fig. 3:**
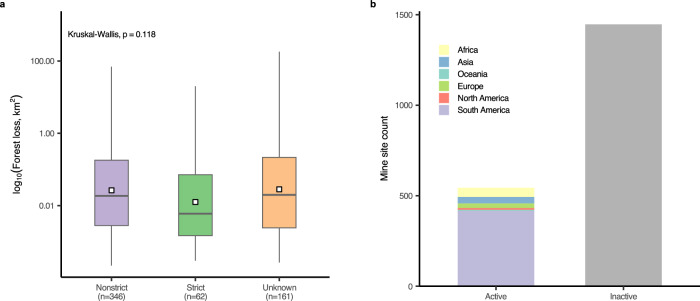
Forest loss and active mine sites within protected areas (PAs). **a** Differences in mining-induced forest loss within PAs classified as Nonstrict, Strict, and Unknown management categories (Two-sided Kruskal-Wallis test, *P* = 0.118). The *y*-axis shows log10-transformed forest loss area. Box represents the interquartile range (IQR, 25th–75th percentiles), the horizontal line indicates the median, the square indicates the mean, and the whiskers extend to the most extreme values within 1.5 × IQR. **b** Number of active and inactive mine sites. Criteria used to classify mine sites as active are described in Methods. Colors indicate the continental distribution of active mine sites. Source data are provided as a Source Data file.

Although Strict PAs exhibited lower forest loss than Nonstrict and Unknown PAs, the differences among PA categories were not statistically significant (Fig. 3a). Forest loss within strict PAs accounted for ~5% (~67 km^2^) of the total loss across all PAs, with the majority concentrated in Asia and South America (Supplementary Fig. 4). Mining activities persisted within PAs, despite their conservation status. Among the mine sites that continuously caused forest loss during the study period, ~27% (544) remained active (Fig. 3b). These active sites resulted in a total forest loss of ~828 km^2^ and were primarily located in South America, followed by Africa and Asia. Brazil hosted the largest number of active mine sites (366), followed by Venezuela (33), Ghana (30), and China (22), whereas all other countries contained fewer than ten sites (Supplementary Table 2). Among active mine sites, the majority (~79%; 429) exhibited accelerating forest loss, with these sites concentrated primarily in Brazil, Ghana, and Venezuela, and more than 300 located in Brazil alone.

### Mining-induced forest loss beyond protected areas

**Fig. 4:**
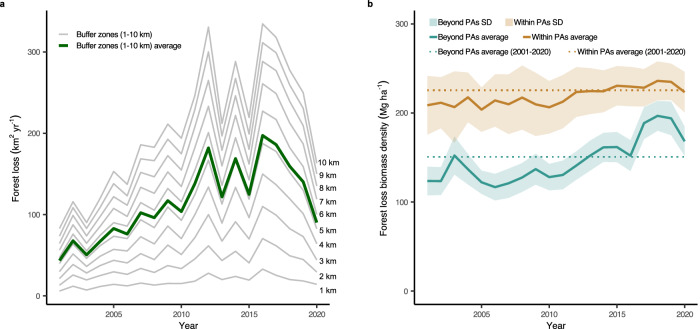
Mining-induced forest loss beyond protected areas (PAs) and associated biomass density changes. **a** Annual mining-induced forest loss within buffer zones extending 1–10 km beyond PA boundaries. Grey lines represent annual forest loss within individual buffer zones, and the green line indicates the average across all buffer zones. **b** Temporal changes in biomass density of forest loss within PAs and their surrounding 10 km buffer zones. Solid lines represent the annual average biomass density, and shaded areas represent average ± 1 standard deviation (SD) across four global biomass datasets. Horizontal dotted lines indicate the average biomass density over the study period (2001–2020). Source data are provided as a Source Data file.

We established buffer zones (1–10 km) outside PA boundaries to analyze mining-induced forest loss beyond PAs. Annual forest loss within the buffer zones surrounding PAs increased significantly at a rate of 9.1 km^2 ^yr^−1^ (MK test, Z = 4.37, *p* < 0.001, 95% CI [6.0, 10.9]; Fig. 4a) before declining after 2016, a pattern consistent with that observed within PAs. Notably, forest loss in the 4 km buffer zones exceeded that within PAs, while forest loss within the 10-km buffer zones reached ~4113 km^2^, nearly three times greater than that within PA boundaries (Supplementary Fig. 5). Similar to patterns observed within PAs, forest recovery beyond PAs remained extremely limited. During 2001–2012, forest gain within the 5 km and 10-km buffer zones accounted for only 3.02% and 3.13% of the corresponding forest loss, respectively (Supplementary Table 1). At the regional scale, forest loss beyond PAs was particularly severe in North America, Asia, and Africa, where forest loss within the 10 km buffer zones was ~28, ~12, and ~7.6 times greater than that within PA boundaries, respectively (Supplementary Fig. 6). At the national scale, countries with substantial forest loss within PA boundaries (e.g., Brazil, Venezuela, Zambia, and Indonesia) also experienced considerable forest loss beyond PAs (Supplementary Table 3). However, some countries with relatively limited forest loss within PAs (e.g., Peru, Russia, Canada, and Australia) exhibited severe forest loss outside PA boundaries, with forest loss within the 10 km buffer zone reaching several-fold, and in some cases hundreds of times, that observed within PA boundaries. Although some mining activities comply with PA regulations (e.g., prohibitions on mining with strict nature reserves), mining-driven forest loss exhibited pronounced spatial expansion along PA edges (Supplementary Fig. 7). This expansion may strengthen the spatial connectivity of forest disturbances inside and outside PAs, thereby increasing pressure on forest conservation within these areas. Moreover, mining activities surrounding different PAs sometimes formed interconnected spatial patterns, which may further undermine the integrity of forest ecosystems at the regional scale.

We also found that the average biomass density increased significantly both within PAs (MK test, Z = 3.53, *p* < 0.001, Sen’s slope=1.47 Mg ha^−1^ yr^−1^, 95% CI [0.99, 1.88]) and within their 10 km buffer zones (MK test, Z = 3.93, *p* < 0.001, Sen’s slope=3.49 Mg ha^−1^ yr^−1^, 95% CI [2.16, 4.89]; Fig. 4b). This trend was particularly pronounced in South America and Asia, regions that harbor some of the world’s largest tropical rainforests. Notably, in South America, the average biomass density of forest loss beyond PAs increased and exceeded that within PA boundaries during the past four years of the study period (Supplementary Fig. 8). Given the higher carbon storage potential of these forests, this trend may exacerbate global carbon loss. We estimated the carbon emissions associated with mining-induced forest loss during the study period as described in Supplementary Note 2. We found that mining-induced forest loss resulted in ~504 ± 22.8 Mt CO_2_ emissions, of which ~13% (~66 ± 3.3 Mt) occurred within PAs and ~30% (~159 ± 7.5 Mt) occurred within their 10 km buffer zones (Supplementary Table 4; Supplementary Fig. 9).

## Discussion

Driven by substantial economic benefits and the growing demand for renewable energy materials, mining has expanded rapidly and become a major driver of deforestation. A considerable proportion of mine sites spatially overlap with PAs^16,17^, raising concerns about their effectiveness in conserving forest ecosystems. This study provides a global-scale quantitative assessment of mining-induced forest loss within and around PAs. Previous studies have primarily relied on spatial analysis of mining activities and PAs to infer potential impacts. For example, one study reported that ~7% of 1,418 mines associated with four key metals directly overlap with PAs, while ~27% are located within 10 km of PA boundaries^16^. Another study, based on ore extraction data from ~3000 mine sites between 2000 and 2019, indicated that 50% of extraction occurred within 20 km of PAs, with 8% (~480 Mt) occurring inside PAs^17^. More recently, a study estimated that over 7000 PAs worldwide have been or are currently affected by mining activities^28^. By integrating global forest change data, mining land-use data, and PA boundary datasets, we further quantify the actual dynamics of forest loss attributable to mining. Our results show that between 2001 and 2020, ~11% (~1,372 km^2^) of global mining-induced forest loss occurred within PAs, while over 30% occurs within a 10 km buffer outside PA boundaries. Although this magnitude of loss may appear modest in a global context, it becomes substantial when considered against the forest area overlapping PAs and mine sites (~3265 km^2^), representing a ~ 42% loss of forest within PAs over the study period (Supplementary Table 5). Our study provides a direct quantification of mining-induced deforestation and, although not strictly comparable with previous assessments, yields results that are broadly consistent with earlier findings, further reinforcing evidence that mining poses a substantial threat to PAs. Our estimates are more conservative than those reported in ref. ^28^, likely due to the use of a more comprehensive global mining inventory in that study (>230,000 mining areas). Given the gaps in global mining datasets^39^, our estimates may underestimate the negative impacts of mining on PAs and even global forests. Moreover, although these studies help identify potential threats posed by mining to PAs, they may not accurately capture the actual deforestation impacts of mining activities. For example, some mine sites that spatially overlap with PAs do not necessarily result in forest loss. We find that the actual area of forest loss within PAs is substantially smaller than the area occupied by mine sites within these areas (Supplementary Table 5). Conversely, some mining activities cause limited forest loss within PA boundaries but may trigger extensive deforestation in surrounding areas (Supplementary Table 3). These findings further highlight that differences in data completeness and methodological frameworks can substantially influence assessments of mining-related threats to PAs.

The ecological footprints and socio-economic drivers of different mining types (e.g., metal versus non-metal mines, artisanal versus industrial) vary considerably^45^, potentially masking heterogeneous impacts on forest loss within PAs. However, the mining land-use datasets used in this study^42,43^ lack sufficient details to support such stratified analyses within our current framework. Given that the rising demand for critical materials under the global energy transition is expected to further accelerate mining expansion, integrating more detailed mining inventories^17,46^ could provide deeper insights into these differences. Moreover, our estimate of the scale and spatiotemporal patterns of mining-induced forest loss is limited to the period 2001–2020 and therefore does not capture more recent developments. Nevertheless, given the projected surge in demand for renewable energy materials and their strong spatial overlap with biodiversity hotspots of conservation importance^17,35,47^, we believe that the conclusions derived from the 20-year analysis remain robust. The standardized analytical framework developed in this study, combined with continuously updated datasets (e.g., GFC v1.12 dataset covering 2001–2024), may provide a valuable foundation for future long-term monitoring and assessment. We also acknowledge that the use of a static biomass map^44^, together with the assumption of constant forest biomass throughout the study period, introduces uncertainties into our estimates. Forest biomass in areas unaffected by mining may have changed over time owing to natural growth or degradation associated with climate variability, edge effects, and other environmental factors^48–51^. Nevertheless, the consistency of results across multiple biomass datasets, together with their similar temporal trends (Supplementary Fig. 10), increases confidence in our findings. Future studies should integrate temporally continuous biomass datasets with high-resolution remote sensing data to more accurately quantify mining-induced biomass loss and improve the precision of these estimates. Finally, we emphasize that the classification of active mine sites in this study is based on detectable forest loss associated with mining expansion. Consequently, some operational mine sites that do not generate additional forest loss, such as those involving underground extraction, expansion within previously cleared areas (e.g., existing pits), or operations located in non-forested landscapes, may have been classified as inactive. However, our analysis specifically focuses on mine sites that have caused sustained forest loss within PAs in recent years. Furthermore, operational mine sites that do not result in additional forest loss are unlikely to exert substantial pressure on forest conservation within or around PAs during the study period. Therefore, our approach provides a conservative estimate of the number of active mine sites and their associated impacts on forest ecosystems.

PAs are being expanded globally to protect forests and biodiversity. However, our results reveal that forests within existing PA boundaries have already experienced extensive degradation associated with mining activities. Although annual mining-induced forest loss has shown a declining trend, deforestation within PAs continues to intensify. These findings underscore the need not only for stricter regulation of mining activities but also for periodic review and adaptive oversight of existing mining concessions within PAs. Moreover, conservation efforts confined to PA boundaries alone are unlikely to effectively mitigate mining-related forest loss, as surrounding areas experience even greater deforestation pressure. Conservation strategies should therefore recognize PAs as integral components of broader ecological networks rather than isolated management units^52,53^. Given that mining impacts can extend tens of kilometers beyond extraction sites^27^, there is an urgent need to establish buffer zones around PAs^54,55^, restore critical ecological corridors^56^, and maintain the connectivity and integrity of PA networks^57^.

Conflicts between conservation and development represent a key constraint on the effectiveness of PAs in protecting forests. In resource-dependent developing countries, blanket prohibitions on mining may face implementation barriers and could limit local communities’ access to land and natural resources. For example, in Brazil, at least 20% of strictly protected areas are currently under consideration for mining development^15,58^. Downgrading or opening existing PAs may further intensify indirect deforestation and forest fragmentation^33^. Therefore, prioritizing the protection of ecologically critical areas while reducing secondary human pressures associated with mining, such as settlement expansion and agricultural development^26^, through land-use alternatives and compensation schemes may offer a viable pathway.

Our findings also highlight the importance of incorporating mining-related forest carbon loss accounting and compensation mechanisms into conservation planning and development decision-making. Mining-driven deforestation increasingly affects forests with higher biomass density both within and beyond PAs, leading to rising carbon emissions per unit area of forest loss. Accordingly, differentiated management strategies across PAs, together with periodic updates to PA classification^59^, may help prioritize high-priority forests and restrict or prohibit new mining activities^60^. Complementary measures, including ecological compensation mechanisms^61^ and post-mining forest restoration requirements^47^, could further ensure that mining operations internalize their ecological and climate responsibilities throughout the entire project lifecycle, thereby mitigating long-term environmental impacts.

## Methods

### Protected areas

We obtained terrestrial protected areas (PAs) from the February 2022 version of the World Database on Protected Areas (WDPA). Because PAs in China and India are partially missing in WDPA versions after 2019^62^, we supplemented these countries using the April 2018 version (Supplementary Fig. 11). As reported by ref. ^63^, this version contains a complete inventory of Chinese PAs available at that time. We did not incorporate additional data from national or regional databases because the WDPA, a joint project of the United Nations Environment Programme (UNEP) and the International Union for Conservation of Nature (IUCN), represents the most comprehensive global database of terrestrial and marine PAs. It is the only authoritative dataset that adheres to consistent global standards and is regularly updated. For PAs with identical WDPAID, we used attribute information (e.g., boundaries, establishment year) from the February 2022 version. PAs present only in the April 2018 version were fully integrated into the 2022 dataset. Following WDPA guidelines and previous global studies^9,64^, we considered only polygon layers and excluded point layers. We excluded PAs without the status “Designated”, “Established”, or “Inscribed”, as well as UNESCO Man and Biosphere Reserves (MAB). For internationally designated PAs (e.g., Ramsar Sites and World Heritage Sites), we adopted the geographic boundaries and IUCN management categories of their corresponding nationally designated PAs to ensure analytical consistency. We also excluded PAs smaller than 900 m^2^ to ensure compatibility with the 30-m resolution Global Forest Change (GFC) dataset. To avoid double-counting geographically overlapping PAs, we performed a dissolve operation on all filtered PA polygons. Spatially overlapping PAs were merged into single polygons, retaining the most restrictive IUCN management category. The IUCN classifies PAs into categories I–VI: Ia (strict nature reserve), Ib (wilderness area), II (national park), III (natural monument or feature), IV (habitat or species management area), V (protected landscape or seascape), and VI (PAs with sustainable use of natural resources), as well as categories without an IUCN assignment (“Not applicable”, “Not assigned”, “Not reported”), which we term “Unknown”. IUCN categories I and II are generally considered strictly PAs with primary biodiversity conservation objectives (Strict), whereas categories III–VI are typically regarded as allowing varying degrees of human activity (Nonstrict).

### Mine site data

We used publicly available global mining land-use datasets^42,43^ (hereafter referred to as Maus’s and Tang’s datasets, respectively) to identify mine sites for this study. Both datasets comprise globally mapped mining polygons derived from manual interpretation of high-resolution satellite imagery and include full surface mining footprints (e.g., waste rock dumps, pits, water ponds, and mining infrastructure). Due to differences in mapping criteria^43^, Tang’s dataset contains a larger number of mining polygons but a smaller total mapped area than Maus’s dataset. To ensure adequate spatial coverage, we used the union of the two datasets for subsequent analyses. Each mine polygon was assigned a unique identifier to track mining activity and its associated forest loss within and around PAs. For overlapping polygons present in both datasets, we used the combined maximum spatial extent and assigned a single ID number (Supplementary Fig. 12).

### Global Forest Change (GFC) data

Mining-induced forest loss during the study period was derived from the Hansen Global Forest Change (GFC) v1.10 dataset^41^, which provides global information on forest cover and forest change at ~30 m spatial resolution. Following previous studies that adopted canopy cover thresholds of 30–50%^65–68^, forest was defined as pixels with tree height exceeding 5 m and canopy cover of at least 40%. Because the GFC dataset does not distinguish between natural forests and plantations, we used the global forest management map^69^ to exclude plantations. This is a 1 km resolution forest management classification map for the year 2000 and includes two levels of forest management, with three categories each. We resampled the “Level 1 layer (Planted)”^69^ to 30 m resolution and overlaid it with the treecover2000 layer from the GFC dataset to remove the influence of plantations. Forest loss occurring within mine polygons was defined as mining-induced direct deforestation. Using the treecover2000 layer as a baseline, we intersected annual forest-loss pixels (2001–2020) from the GFC dataset with mine polygons to estimate annual forest loss attributable to mining. Forest gain was not considered because the GFC dataset does not provide annual observations of forest gain.

### Quantifying forest loss within and beyond protected areas

We quantified forest loss within PAs by spatially overlaying annual mining-induced forest loss with PA polygons. Calculating forest loss across the entire study period for all PAs could overestimate mining impacts because a substantial proportion of PAs were established after 2000. Unlike many previous studies, we did not exclude PAs established after 2000, as this would result in data loss and potentially underestimate the impacts of mining (Supplementary Fig. 13). Instead, we employed the following approach: (i) for PAs established before 2000, we calculated forest loss over the entire study period (2001–2020), and (ii) for PAs established after 2000, we calculated forest loss from their year of establishment (STATUS_YR) to 2020. To assess the contribution of mining to forest loss within PAs, we calculated an annual indicator, Proportion_loss (%), defined as the proportion of mining-induced forest loss occurring within PAs relative to total global mining-induced forest loss:

1 $${{\rm{Proportion}}}\_{{{\rm{loss}}}}_{{{\rm{i}}}}=\frac{{{\rm{Forest}}}\; {{\rm{loss}}}({{\rm{PA}}}{{{\rm{s}}}})_{{{\rm{i}}}}}{{{\rm{Total}}}\; {{\rm{forest}}}\; {{\rm{lo}}}{{{\rm{ss}}}}_{{{\rm{i}}}}}$$

where Forest loss (PAs)_i_ and ${{\mbox{Total forest loss}}}_{{\mbox{i}}}$ represent mining-induced forest loss within PAs and total global mining-induced forest loss, respectively, for a given year *i* (from 2001 to 2020).

We also quantified forest loss beyond PA boundaries, as external pressures often serve as early warning indicators for internal forest loss. Specifically, we established buffer zones at 1 km intervals extending from 1 to 10 km beyond each PA boundary and calculated mining-induced forest loss within each buffer zone to assess mining impacts beyond PAs.

### Classifying active mine sites

Identifying mine sites that continue to expand and drive forest loss is essential for understanding the long-term pressure of mining on forests, particularly for those that have already caused forest loss within PAs. Such sites may continue to threaten forests both within and beyond PA boundaries. We therefore first identified all mine sites associated with forest loss within PAs during the study period and subsequently assessed their activity status. Because the global mining datasets used in this study do not provide information on operational status or activity timelines, we developed an approach based on the temporal dynamics of forest loss. Here, active mine sites were defined as those exhibiting continued expansion and detectable forest loss during the study period (Supplementary Fig. 14). Specifically, mine sites were classified according to temporal trajectories of mining-induced forest loss from 2001 to 2020 using the following criteria: (i) If no forest loss was observed between 2001 and 2017, but forest loss occurred in three consecutive years (2018–2020), the mine site was classified as active. This criterion likely identifies newly established mines or mines that only recently became operational. We selected a three-year window to reflect the typically continuous nature of mining activities and to reduce intra-annual errors in the GFC dataset. (ii) If forest loss was observed between 2001 and 2017, and cumulative forest loss during 2018–2020 accounted for more than 20% of total forest loss during 2001–2020, the mine site was classified as active. This criterion likely identifies mines that remained operational throughout the study period and continued to expand in recent years. Based on these criteria, mine sites were classified as either active or inactive. For each identified active mine, we further fitted a linear regression model to annual forest loss to assess whether forest loss was accelerating. Notably, this approach was designed to identify mine sites associated with ongoing forest loss. Because activity classification relied on detectable forest loss, it was unlikely to capture mine sites that remained operational but did not generate additional forest loss.

### Temporal analysis of forest biomass density

We used the global aboveground live woody biomass density map^44^ (hereafter referred to as Harris’s biomass map) to analyze temporal dynamics in biomass density associated with forest loss within PAs and their surrounding 10 km buffer zones. Harris’s biomass map is a global map at 30 m spatial resolution for the year 2000, generated by integrating the approaches developed by refs. ^70,71^. It integrates LiDAR-derived biomass estimates and regional allometric equations from more than 700,000 field plots with a random forest model trained on Landsat imagery. Because this map represents a static estimate for the year 2000, we assumed that forest biomass in areas unaffected by mining remained constant throughout the study period (2001–2020). We overlaid annual mining-induced forest loss within PAs and their 10 km buffer zones onto the biomass map and calculated the average biomass density at the pixel scale. To assess potential uncertainty associated with biomass estimates, we incorporated three additional global datasets, including the GEOCARBON biomass map, the Harmonized above/belowground biomass map, and the ESA CCI biomass map. The GEOCARBON biomass map^72^ provides global aboveground biomass estimates for the year 2000 at a spatial resolution of 0.01°, which integrates a pantropical biomass dataset^72^ with a boreal forest biomass component^73^. The Harmonized above/belowground biomass map^74^ (hereafter referred to as Spawn’s biomass map) provides harmonized global above/belowground biomass carbon density for 2010 at 300 m resolution and incorporates remote sensing products representing woody vegetation, grasslands, croplands, and tundra. The ESA CCI global biomass dataset^75^ (v5.01) provides aboveground biomass estimates for 2010 and 2015–2021 at 100 m spatial resolution. This product integrates multiple Earth observation datasets, including Sentinel-1, ALOS-1, ALOS-2, Envisat ASAR, and optical imagery, using ensemble algorithms to ensure interannual consistency. We used the 2010 ESA CCI biomass map to align temporally with the Spawn’s biomass map. At the annual scale, we calculated the standard deviation (SD) of biomass density associated with forest loss across the four biomass datasets and used it as an uncertainty estimate for the annual average biomass density derived from Harris’s biomass map (Fig. 4b). Temporal trends were also evaluated independently for each biomass dataset to assess the consistency of results across datasets (Supplementary Fig. 10). Because the ESA CCI and Spawn’s biomass maps represent conditions in 2010, biomass values for forest loss occurring during 2001–2009 were extracted from the nearest neighboring pixels.

### Statistical analysis

For time series analyses of mining-induced forest loss from 2001 to 2020, including total forest loss, the proportion of forest loss occurring within PAs, and average biomass density of forest loss, we used a non-parametric Theil-Sen estimator^76^ to assess temporal trends and estimate 95% confidence intervals. Statistical significance was tested using the Mann-Kendall test^77^. To evaluate the influence of PA management categories on forest conservation, we performed a Kruskal-Wallis test^78^ to assess differences in mining-induced forest loss among different strictness levels (Strict, Nonstrict, and Unknown).

### Reporting summary

Further information on research design is available in the Nature Portfolio Reporting Summary linked to this article.

## Supplementary information

Supplementary Information

Peer Review file

Reporting Summary

## Source data

Source Data

## Data Availability

Protected areas (PAs) are obtained from the February 2022 version of the World Database on Protected Areas (WDPA, https://www.protectedplanet.net/en/). The Hansen Global Forest Change (GFC, v1.10) dataset is available at https://storage.googleapis.com/earthenginepartners-hansen/GFC-2022-v1.10/download.html. The Maus’s (Version 2) and Tang’s global mining land-use datasets are available at https://doi.pangaea.de/10.1594/PANGAEA.942325, and https://zenodo.org/records/6806817, respectively. The 2000 global forest management map is available at https://www.environmentalgeography.nl/site/data-models/data/forest-classes-and-uses/. The global aboveground live woody biomass density map is available at https://data.globalforestwatch.org/datasets/gfw::aboveground-live-woody-biomass-density/about. The GEOCARBON global aboveground forest biomass is available at 10.5281/zenodo.20534466. The Harmonized above/belowground biomass carbon density map can be accessed from https://developers.google.com/earth-engine/datasets/catalog/NASA_ORNL_biomass_carbon_density_v1?hl=zh-cn. The ESA CCI global above-ground biomass (v5.01) can be accessed from https://gee-community-catalog.org/projects/cci_agb/?h=esa+cci+agb.#dataset-preprocessing-for-gee. Source data are provided with this paper.

## Code availability

The code used to generate the results presented in this study is publicly available at (https://zenodo.org/records/20553030). (10.5281/zenodo.20553029).
