# Supplementary material for: Global mining has undermined forest conservation within and beyond protected areas
Source: Nat Commun. 2026 Jun 24;17:7954. doi: 10.1038/s41467-026-74859-3 (PMC13448669; doi:10.1038/s41467-026-74859-3)
Supplement: Supplementary file 1 [file 41467_2026_74859_MOESM1_ESM.pdf]

Supplementary Information for

Global mining has undermined forest conservation within and beyond protected areas

He Ren[1,†], Yihua Hu[2,†], Tingting He[3,4,5,*], Yanling Zhao[5], Youpeng Lu[4], Fashuai Li[3], Maoxin Zhang[4], Jianyong Zhang[5], Hongtao Cao[1], Huicai Yang[1], Jin Wu[6], Zhipei Sun[7], Jianyu Wang[8], Yuwei Chen[8,3]

[1]Academy of Eco-Civilization Development for Jing-Jin-Ji Megalopolis, Tianjin Normal University, Tianjin 300387, PR China

[2]State Key Laboratory of Pulsed Power Laser Technology, Hefei 230000, PR China

[3]Advanced Laser Technology Laboratory of Anhui Province, Hefei 230000, PR China

[4]Department of Land Management, Zhejiang University, Hangzhou 310058, PR China

[5]Institute of Land Reclamation and Ecological Restoration, China University of Mining and Technology (Beijing), Beijing 100083, PR China

[6]Advanced Interdisciplinary Institute of Satellite Applications, Beijing Normal University, Beijing 100875, PR China

[7]Department of Electronics and Nanoengineering and QTF Centre of Excellence, Aalto University, Aalto FI-00076, Finland

[8]Hangzhou Institute of Advanced Studies, University of Chinese Academy of Science, Hangzhou 310024, PR China

[†] These authors contributed equally: He Ren, Yihua Hu

*Corresponding author: Tingting He (tthe@zju.edu.cn)

This PDF file includes

Supplementary Note 1 to Note 2

Supplementary Fig. 1 to Fig. 14

Supplementary Table 1 to Table 5

Supplementary References

This document contains the following:

Supplementary Note 1: Post-mining forest gain within and beyond protected areas (PAs).....3
Supplementary Note 2: Estimating CO_2 emissions from mining-induced forest loss 4
Supplementary Fig. 1. Annual mining-induced forest loss across continents from 2001 to 2020 6
Supplementary Fig. 2. Annual mining-induced forest loss within protected areas (PAs) across continents from 2001 to 2020 7
Supplementary Fig. 3. Annual Proportion_loss across continents from 2001 to 2020 8
Supplementary Fig. 4. Distribution of mining-induced forest loss within protected areas (PAs) under different management categories........9
Supplementary Fig. 5. Mining-induced forest loss in buffer zones surrounding protected areas (PAs) 10
Supplementary Fig. 6. Comparison of mining-induced forest loss within and beyond protected areas (PAs) across continents 11
Supplementary Fig. 7. Spatiotemporal patterns of mining-induced forest loss beyond protected areas (PAs) 12
Supplementary Fig. 8. Temporal trends in biomass density of forest loss both within and beyond protected areas (PAs)........13
Supplementary Fig. 9. Annual CO_2 emissions from mining-induced forest loss from 2001 to 2020 14
Supplementary Fig. 10. Consistency analysis of temporal trends in biomass density of forest loss 15
Supplementary Fig. 11. Comparison of protected areas (PAs) in China and India across different WDPA versions 16
Supplementary Fig. 12. Schematic illustration of mine site identification..............................17
Supplementary Fig. 13. Mining-induced forest loss estimates based on different protected areas (PAs) processing approaches........18
Supplementary Fig. 14. Conceptual diagram of active mine site classification......................19
Supplementary Table 1. Post-mining forest gain statistics 20
Supplementary Table 2. Active mine sites within protected areas (PAs) at the national scale 21
Supplementary Table 3. Forest loss within and beyond protected areas (PAs) at the national scale 23
Supplementary Table 4. Total CO_2 emission associated with mining-induced forest loss from 2001–2020........25
Supplementary Table 5. Global distribution of forest cover, mine sites, and their overlap with protected areas (PAs) 26
Supplementary References........27

Supplementary Note 1: Post-mining forest gain within and beyond protected areas (PAs)

We quantified post-mining forest recovery using the Hansen Global Forest Change (GFC, v1.10) dataset[1]. Considering that the GFC dataset does not provide annual information on forest gain but only reports the cumulative value from 2001 to 2012, we also calculated the cumulative forest loss over the same period to ensure analytical consistency. Given that forest recovery generally occurs after mining-induced disturbance, we calculated cumulative forest loss during 2001–2010 to allow sufficient time for subsequent forest gain to be detected within the 2001–2012 forest gain layer. We quantified forest recovery within PAs and in the 5-km and 10-km buffer zones outside their boundaries and use a simple ratio of cumulative forest gain to cumulative forest loss to represent forest recovery during this period (Supplementary Table 1). The Recovery metric was calculated as follows:

$$\text{Recovery} = \frac{\text{Total\_gain\_area}}{\text{Total\_loss\_area}} \quad (1)$$

where Recovery (%) represents post-mining forest recovery during 2001–2012, with higher values indicating better recovery. Total_gain_area denotes the cumulative forest gain from 2001 to 2012, while Total_loss_area represents the cumulative forest loss from 2001 to 2010.

Supplementary Note 2: Estimating CO_2 emissions from mining-induced forest loss

We estimated carbon emissions from mining-induced forest loss during 2001–2020, focusing on aboveground biomass carbon (AGC), belowground biomass carbon (BGC), and soil organic carbon (SOC). Our analysis exclusively addressed carbon loss and did not account for potential carbon gains from post-mining reforestation or natural regrowth. To estimate AGC, we spatially overlaid mining-induced forest loss with a global biomass map[2] and applied a stoichiometric factor to derive pixel-scale carbon stocks from aboveground biomass. Given that the stoichiometric factor typically ranges from 0.45 to 0.50 (e.g., 0.45[3,4], 0.47[5], 0.49[6], 0.50[6,7,8]), following Koh et al. (2021)[9], we calculated results using three stoichiometric factors (0.45, 0.475, and 0.50) and report the average ± standard deviation (SD) as the final estimate. Belowground biomass (BGB) was estimated at the pixel-scale and convert to carbon values using the same three stoichiometric factors. The belowground biomass was calculated by[10], as follows:

$$\mathrm{BGB} = 0.489 \times \mathrm{AGB}^{0.89} \tag{2}$$

where BGB denotes belowground biomass and AGB denotes aboveground biomass. Mining often removes surface vegetation and soil, leading to SOC loss. Therefore, we estimated mining-induced SOC loss using the 0–30 cm topsoil layer from the European Soil Data Centre[11], as follows:

$$\mathrm{SOC} = \mathrm{OCS} \times \theta \tag{3}$$

where SOC is soil organic carbon loss due to mining-induced forest loss (Mg C ha^{-1}), OCS is SOC stocks (0–30 cm depth), and θ is the SOC loss rate.

The original dataset was resampled to 30-m resolution using the nearest neighbor method to match the spatial resolution of GFC data. SOC loss rate is largely determined by land-use type following forest loss. Here we referred to a tropical meta-analysis[12] that provides SOC loss rates (0–30 cm) when forest (primary and secondary) is converted to cropland (~20%), grassland (~10%), and secondary forest (~9%). Given the extremely limited post-mining forest recovery, we used an average value (~15%) of the cropland and grassland conversion rates to estimate mining-

related SOC loss. Finally, we applied a conversion factor of 3.67 to convert AGC, BGC, and SOC into CO_2-equivalent emissions. We quantified carbon emissions at both global and continental scales, within PAs, and within the 10-km buffer zones outside PA boundaries (Supplementary Table 4), and further assessed their annual trends (Supplementary Fig. 9).

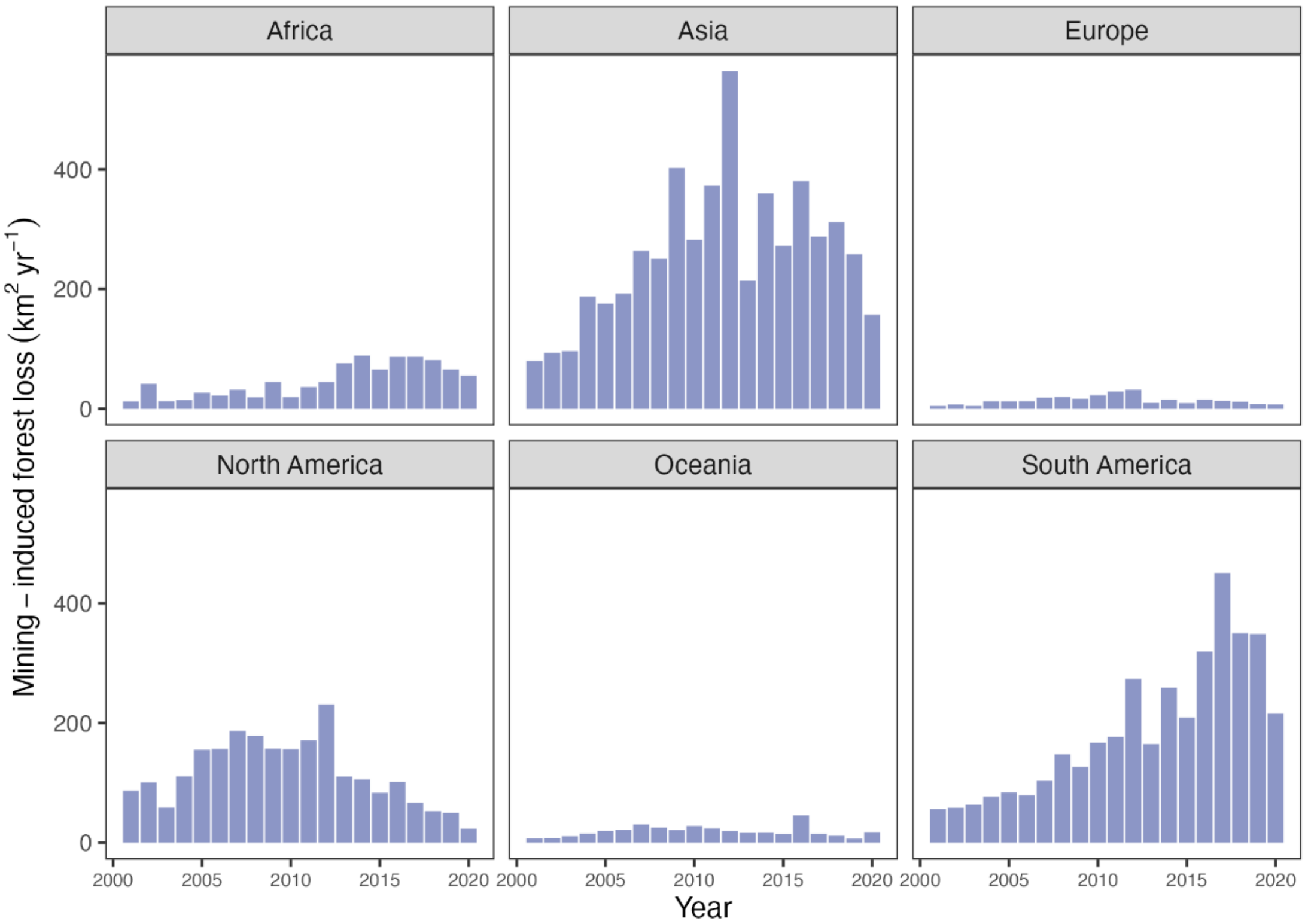

Supplementary Fig. 1. Annual mining-induced forest loss across continents from 2001 to 2020

Source data are provided as a Source Data file.

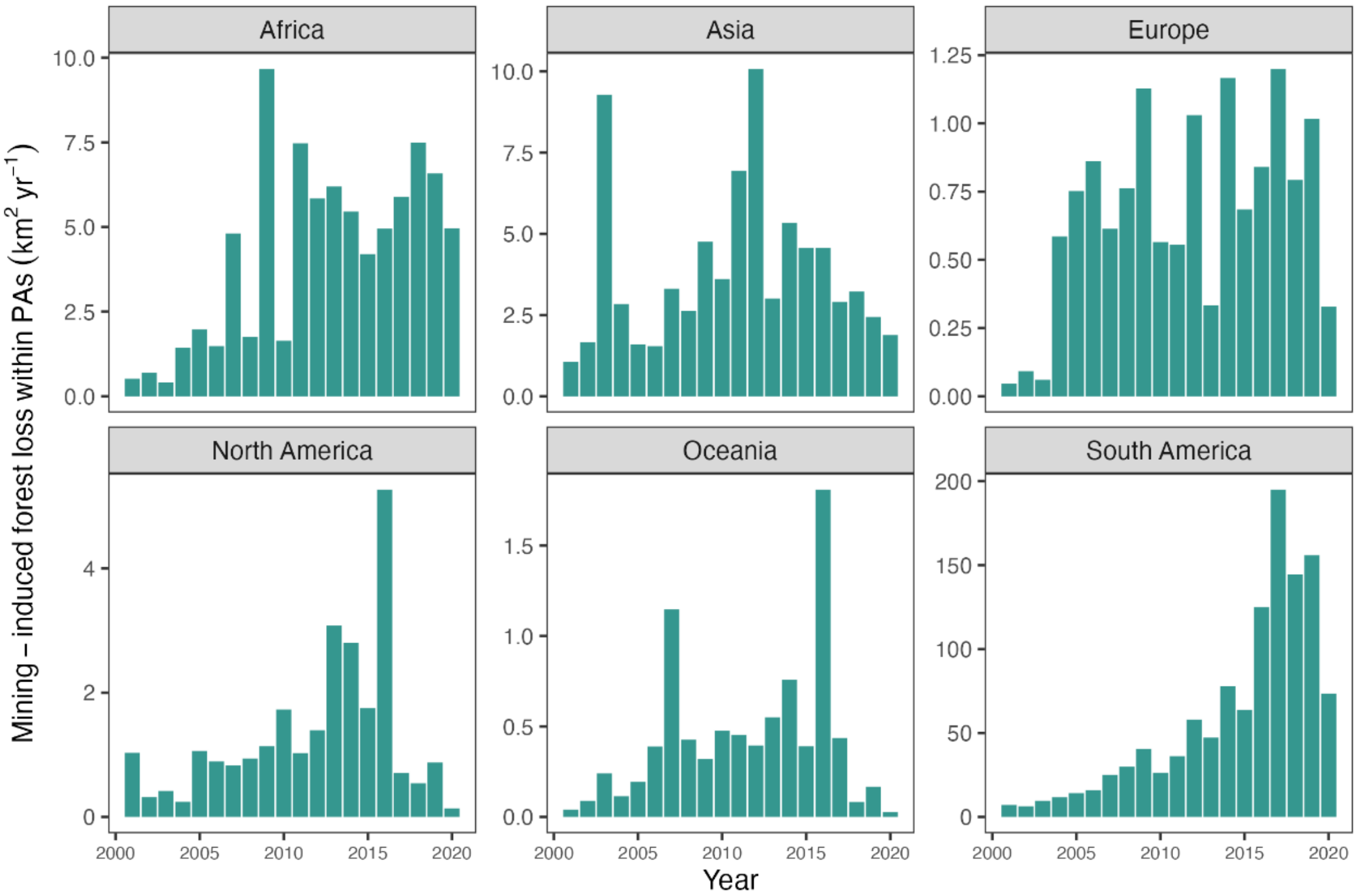

Supplementary Fig. 2. Annual mining-induced forest loss within protected areas (PAs) across continents from 2001 to 2020

Source data are provided as a Source Data file.

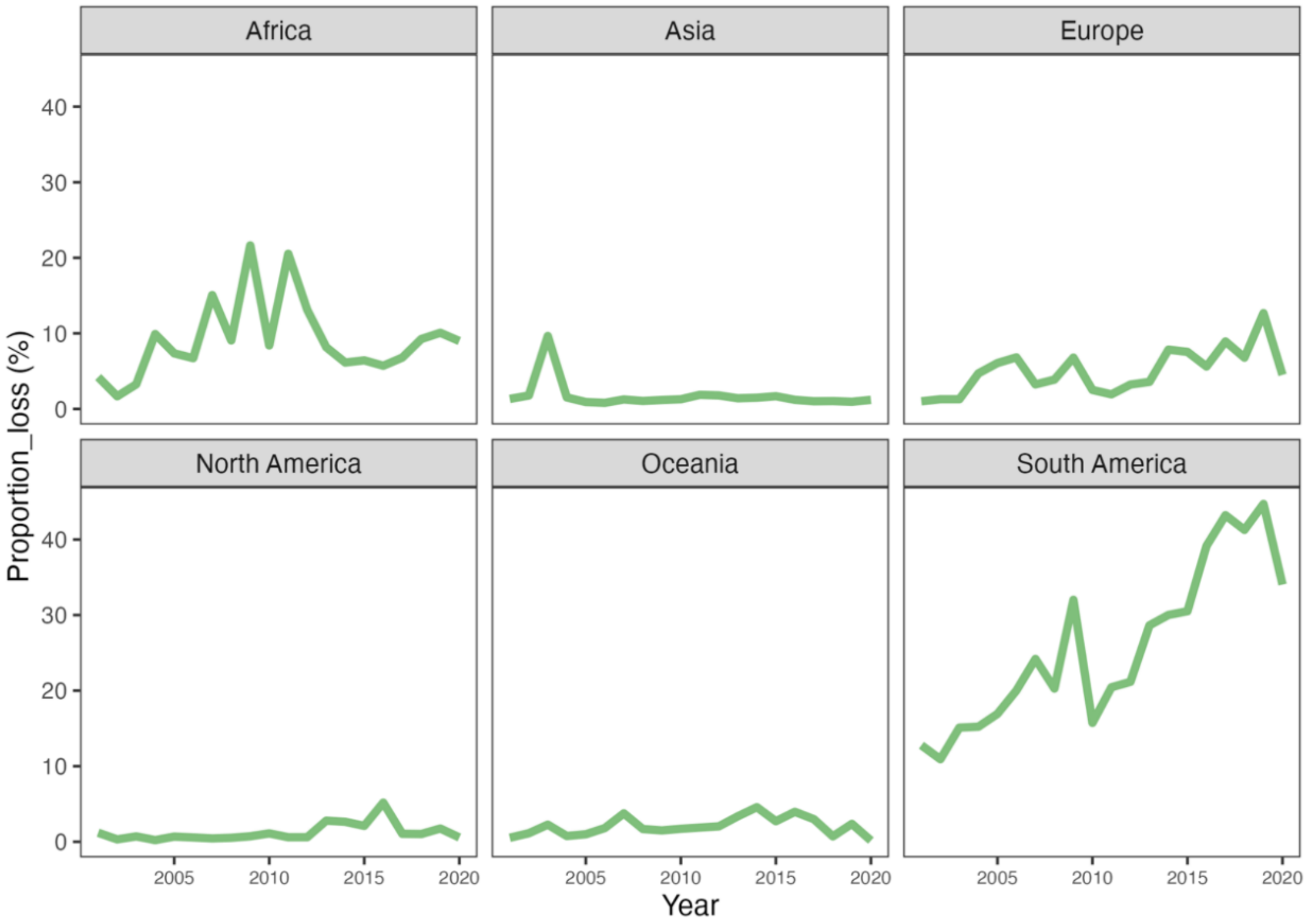

Supplementary Fig. 3. Annual Proportion_loss across continents from 2001 to 2020

Proportion_loss is defined as the proportion of mining-induced forest loss occurring within protected areas (PAs) relative to total global mining-induced forest loss. Source data are provided as a Source Data file.

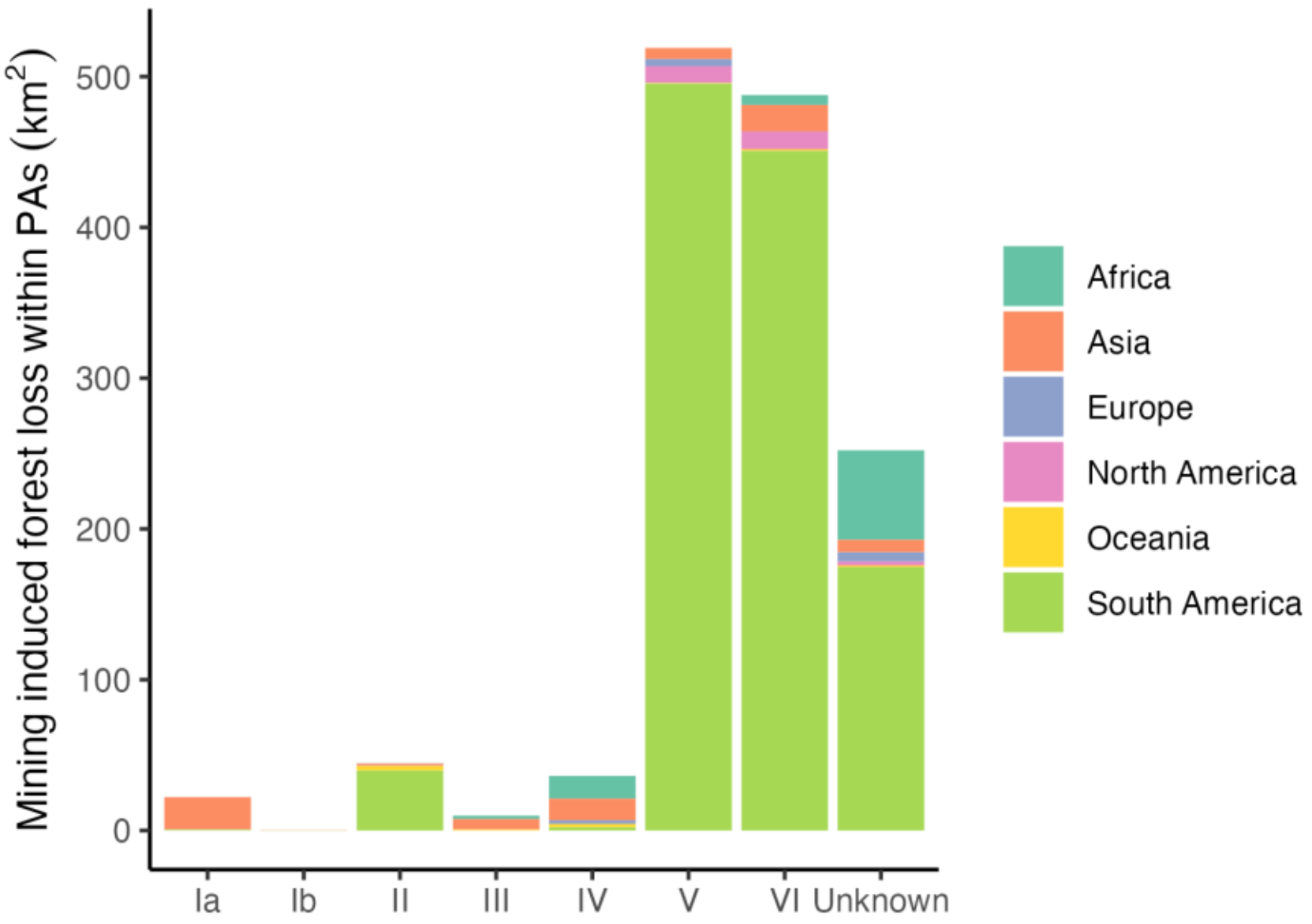

Supplementary Fig. 4. Distribution of mining-induced forest loss within protected areas (PAs) under different management categories

The International Union for Conservation of Nature (IUCN) classifies protected areas (PAs) into management categories Ia—VI, Ia (strict nature reserve), Ib (wilderness area), II (national park), III (natural monument or feature), IV (habitat or species management area), V (protected landscape or seascape), and VI (PAs with sustainable use of natural resources). PAs without an IUCN assignment ("Not applicable", "Not assigned", "Not reported") are grouped as "Unknown" in the figure. Source data are provided as a Source Data file.

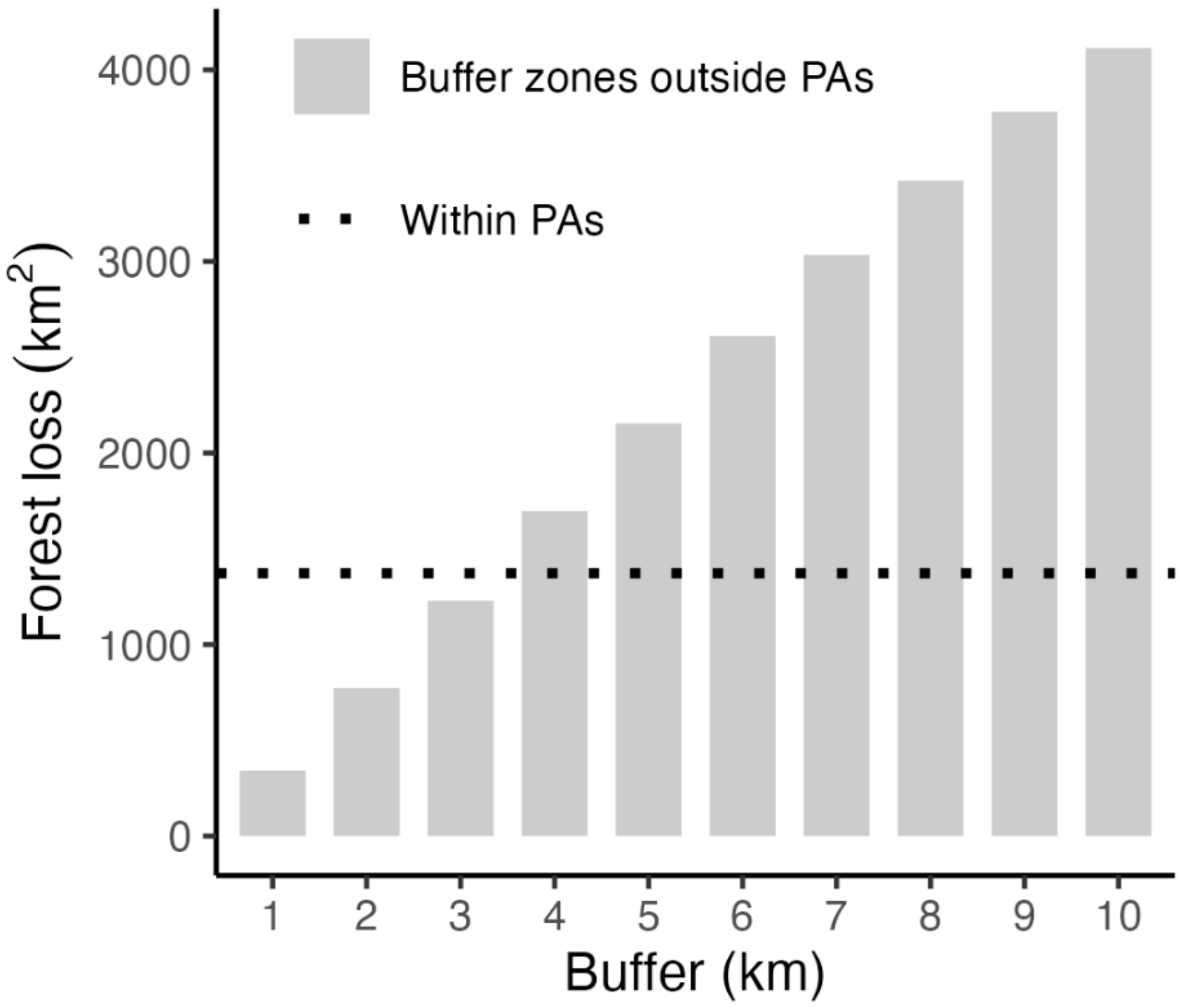

Supplementary Fig. 5. Mining-induced forest loss in buffer zones surrounding protected areas (PAs)

Bars indicate the total area of mining-induced forest loss within different buffer zones (1 to 10 km) outside PA boundaries. The dotted line indicates the total area of mining-induced forest loss occurring within PAs. Source data are provided as a Source Data file.

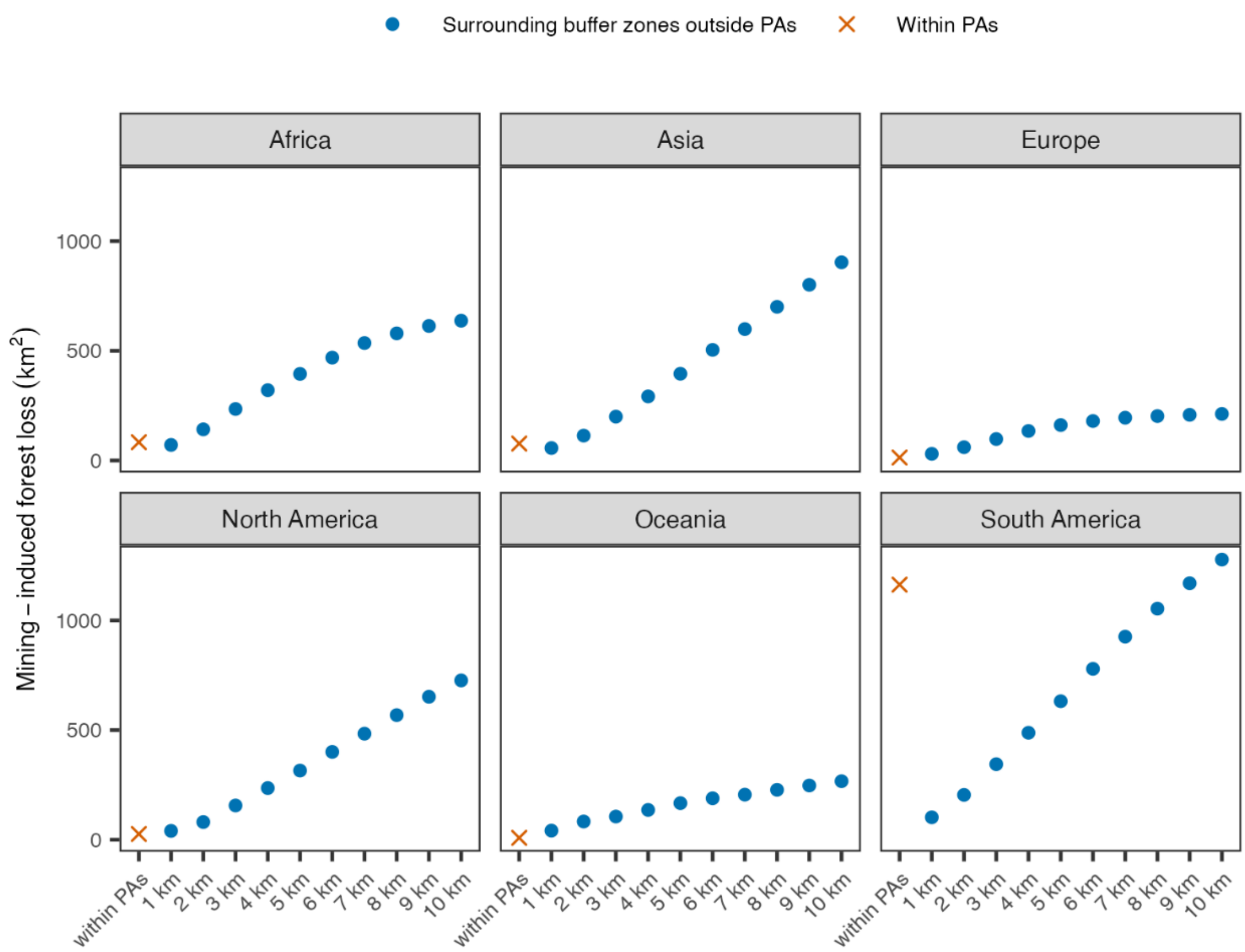

Supplementary Fig. 6. Comparison of mining-induced forest loss within and beyond protected areas (PAs) across continents

Orange crosses and blue circles indicate the total area of mining-induced forest loss within PAs and different buffer zones outside PAs from 2001 to 2020. Source data are provided as a Source Data file.

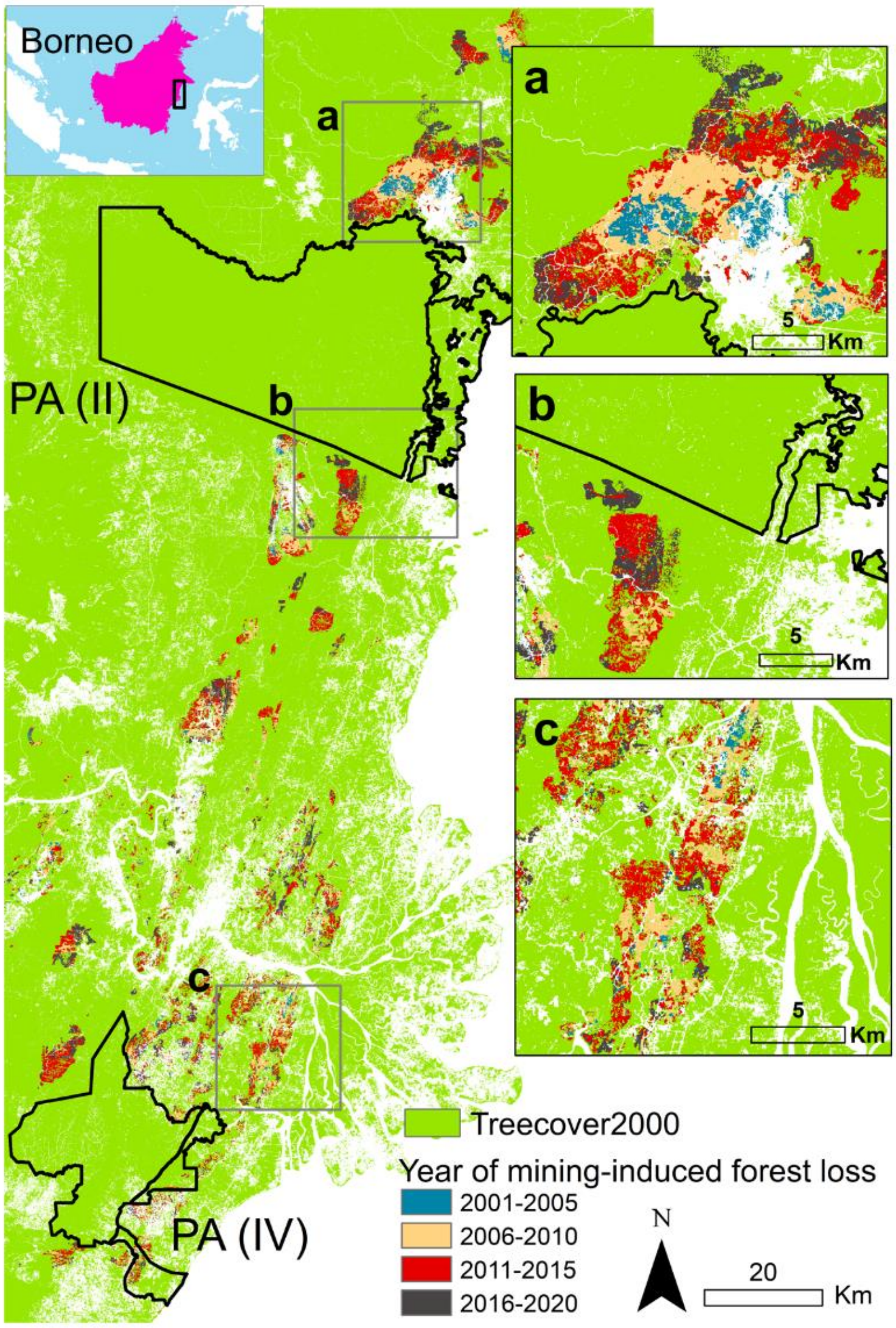

Supplementary Fig. 7. Spatiotemporal patterns of mining-induced forest loss beyond protected areas (PAs)

The figure shows two protected areas (PAs, IUCN categories II and VI) in Kalimantan, Indonesia. Forest cover in 2000 (Treecover2000) and year of mining-induced forest loss (2001–2020) are displayed at 30-m resolution, based on the GFC dataset[1]. **a**, **b** show mining expansion towards PA boundaries, resulting in increasing loss in adjacent forests. **c** shows extensive mining expansion outside PA boundaries, leading to the development of connected areas of forest loss surrounding the protected area. The distribution of forest loss was created using ArcGIS v10.2 software and the underlying world map is sourced from https://www.naturalearthdata.com/. Source data are provided as a Source Data file.

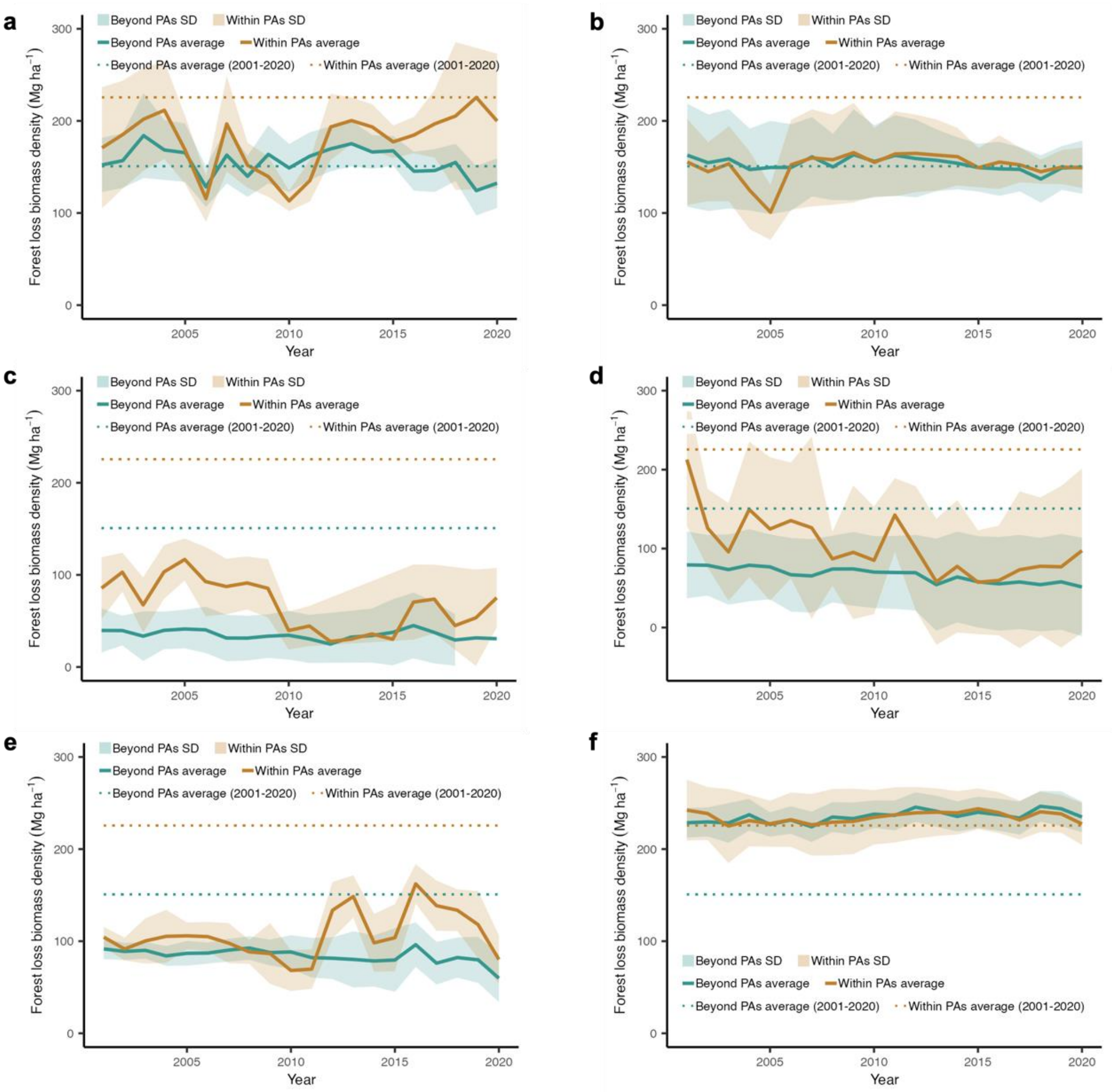

Supplementary Fig. 8. Temporal trends in biomass density of forest loss both within and beyond protected areas (PAs)

a Asia. **b** Africa. **c** Europe. **d** Oceania. **e** North America. **f** South America. Solid lines represent the annual average biomass density from the biomass map of Harris et al.[2]. Shaded areas represent ± 1 standard deviation (SD) calculated across four global biomass datasets[2,13,14,15]. Horizontal dotted lines indicate average biomass density over the period (2001–2020). Source data are provided as a Source Data file.

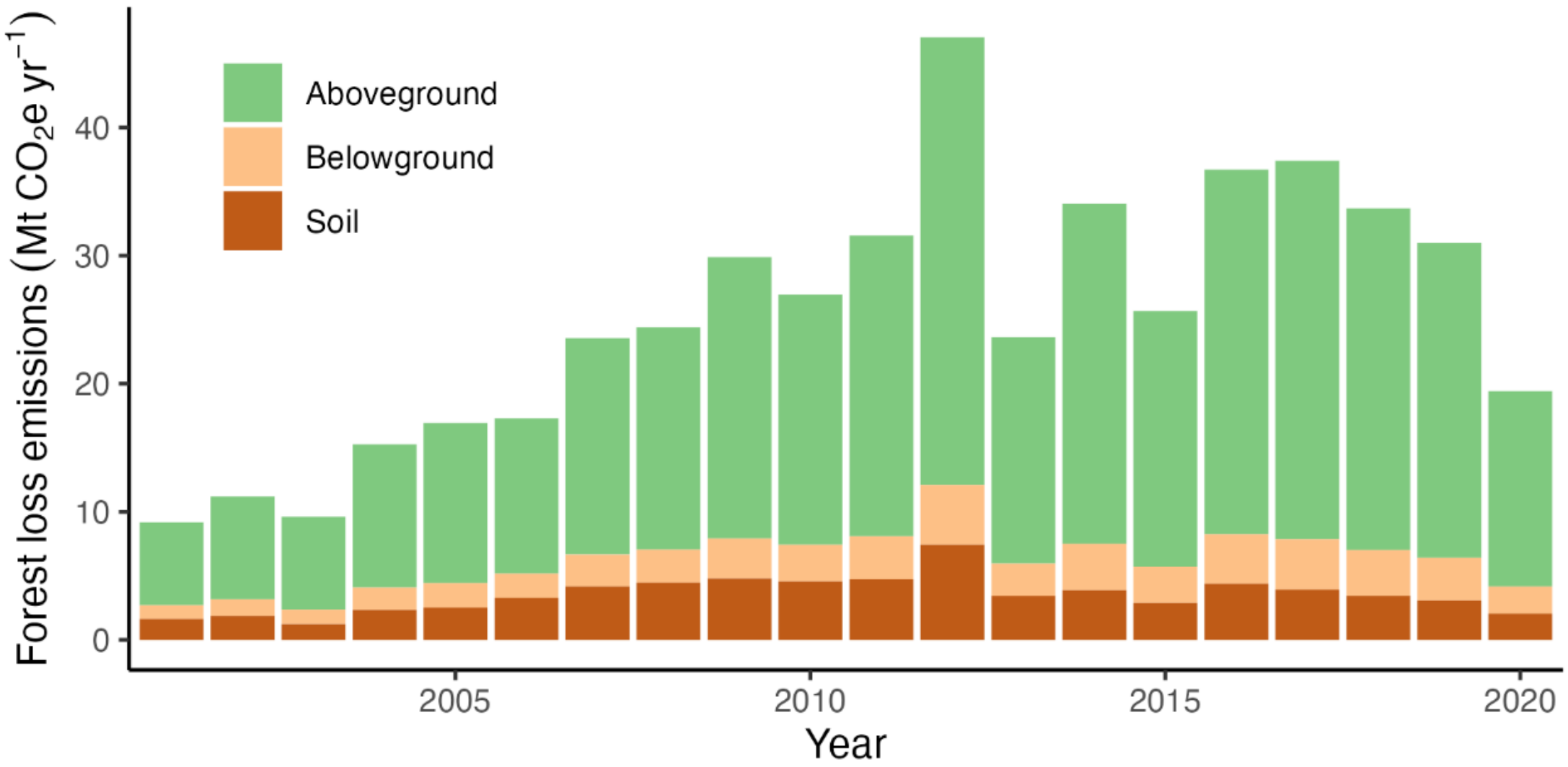

Supplementary Fig. 9. Annual CO_2 emissions from mining-induced forest loss from 2001 to 2020

Carbon emissions were calculated as the sum of soil organic carbon (Soil), average aboveground biomass carbon (Aboveground), and average belowground biomass (Belowground). Calculation details can be found in Supplementary Note 2. Source data are provided as a Source Data file.

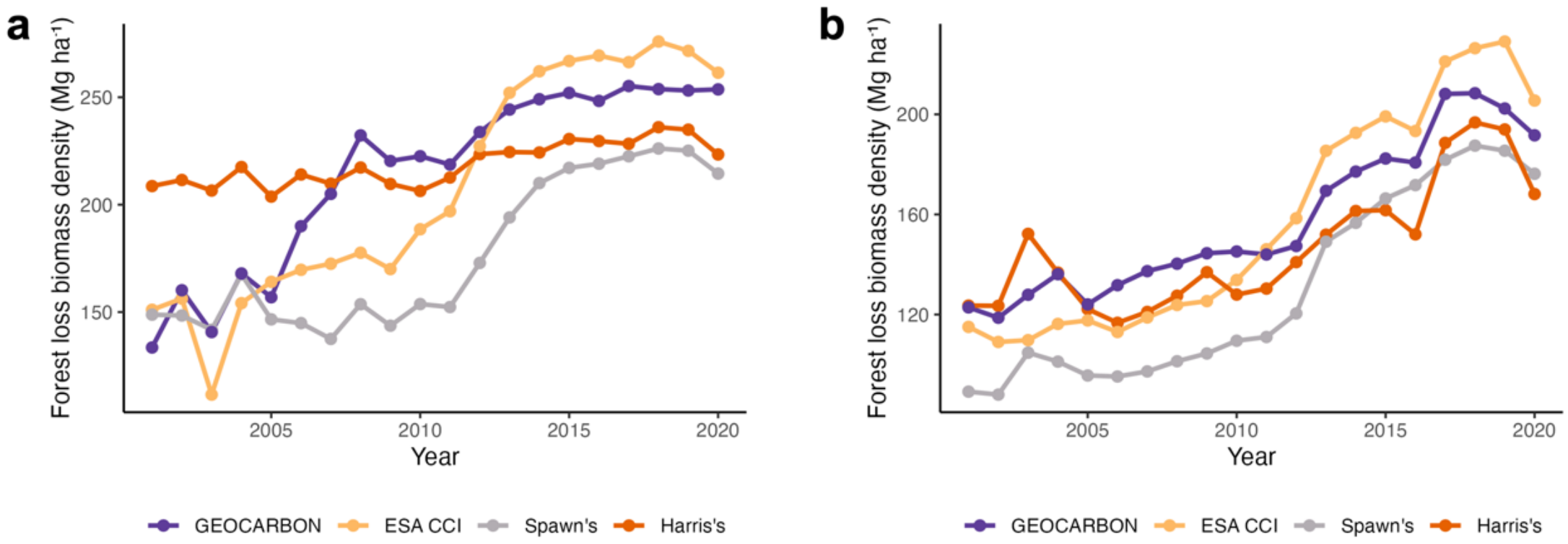

Supplementary Fig. 10. Consistency analysis of temporal trends in biomass density of forest loss

a Within protected areas (PAs). **b** 10-km buffer zones outside PAs. Temporal trends in biomass density of mining-induced forest loss are evaluated across different biomass datasets (Harris's[2], GEOCARBON[13], ESA CCI[14], and Spawn's[15]). Source data are provided as a Source Data file.

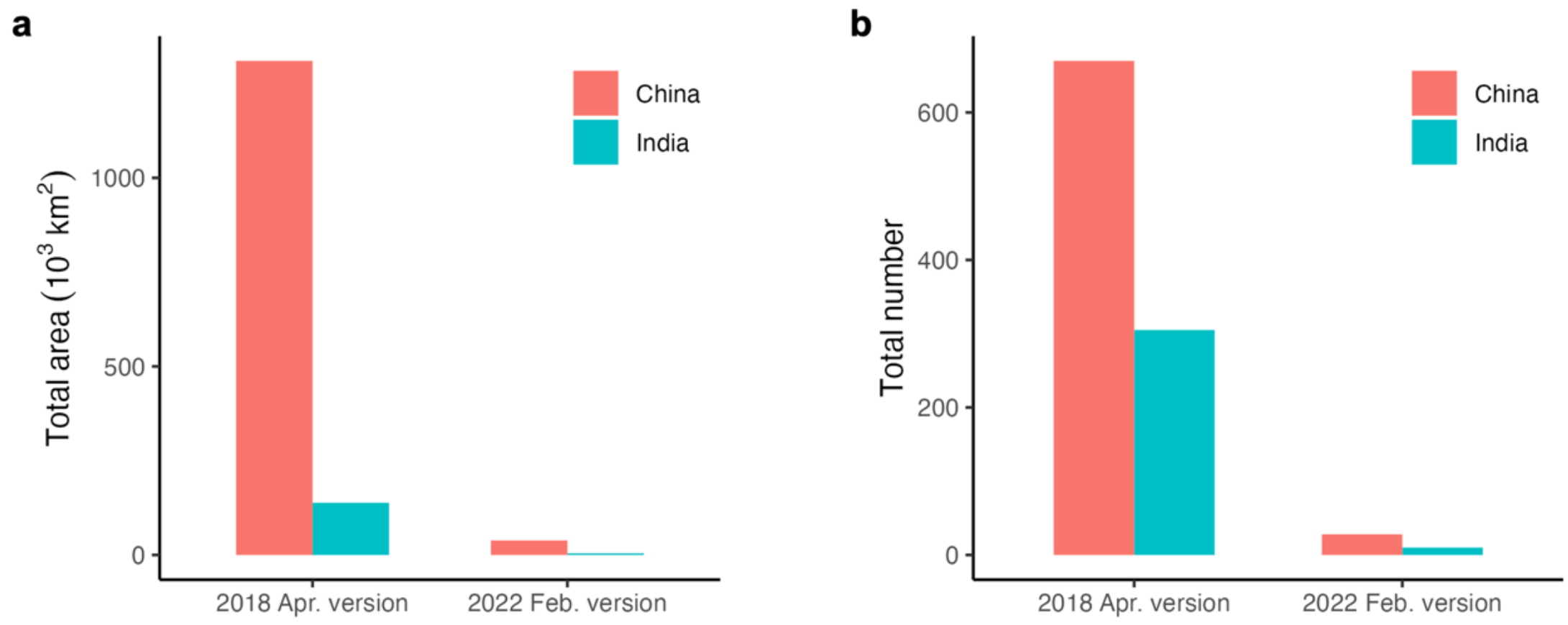

Supplementary Fig. 11. Comparison of protected areas (PAs) in China and India across different WDPA versions

a Comparison of the total PA area (km^2). **b** Comparison of the total number of PAs. PAs was obtained from the February 2022 version (2022 Feb. version) and the April 2018 version (2018 Apr. version) of the World Database on Protected Areas (WDPA, https://www.protectedplanet.net/en/). Source data are provided as a Source Data file.

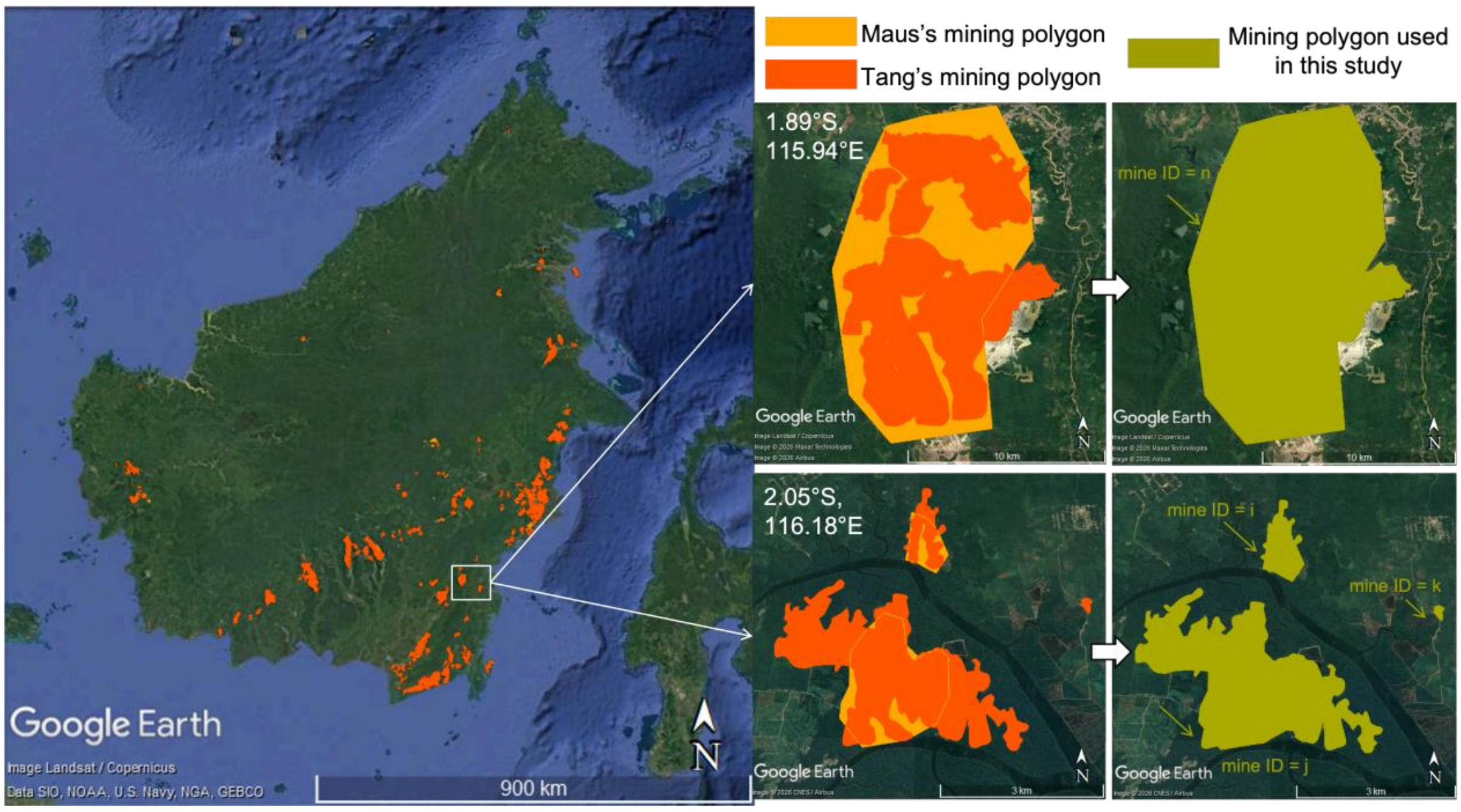

Supplementary Fig. 12. Schematic illustration of mine site identification

The figure shows representative mine sites (Kalimantan) overlaid on Google Earth Satellite Imagery (Map data: Google Earth, © Maxar Technologies). The mine sites used in this study are derived from the union of Maus's[16] and Tang's[17] global mine land-use datasets. For mine sites with spatial overlap between the two datasets, the maximum spatial extent was retained to define a single mine site, and a unique ID number was assigned.

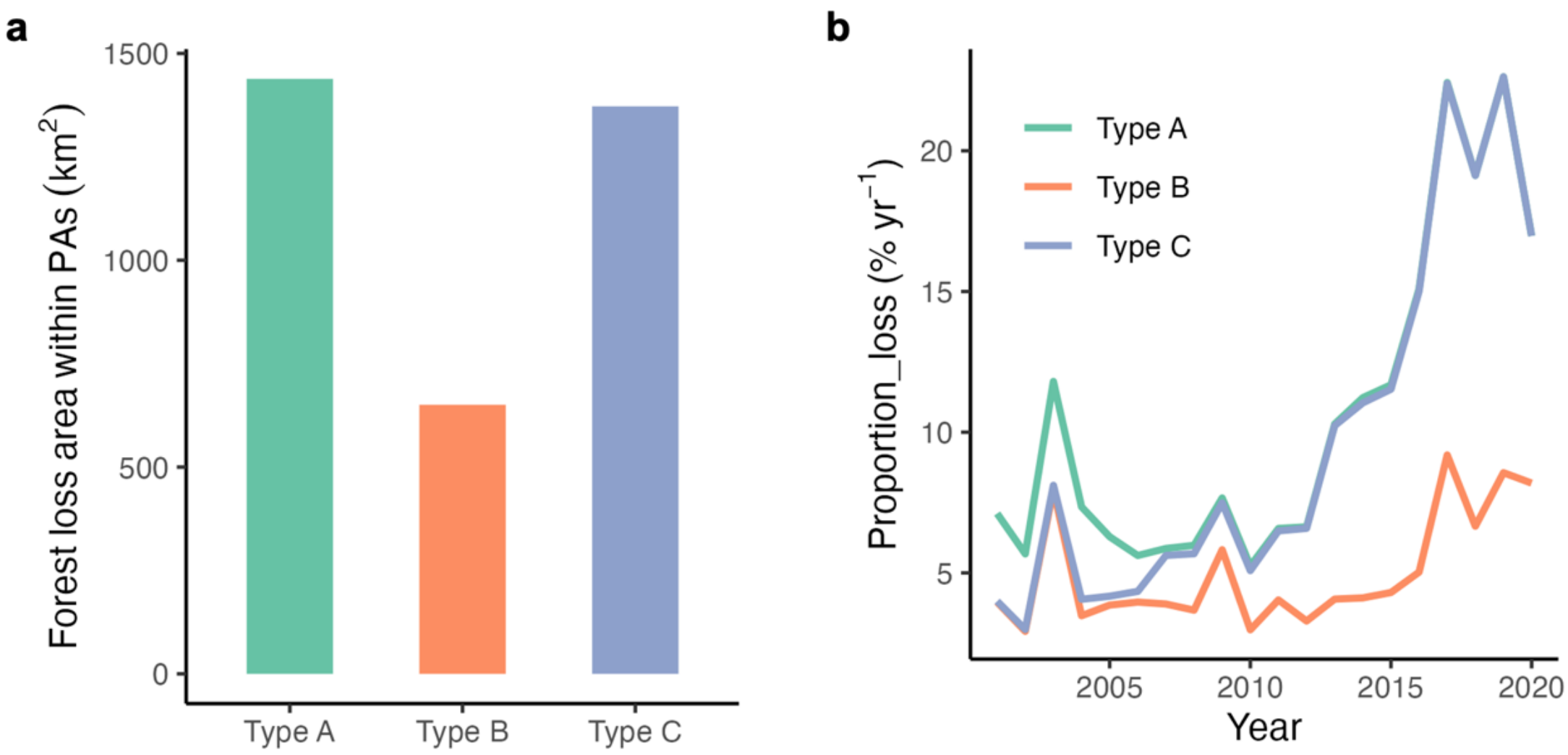

Supplementary Fig. 13. Mining-induced forest loss estimates based on different protected areas (PAs) processing approaches

a Total mining-induced forest loss within PAs from 2001 to 2020. **b** Temporal trends of Proportion_loss from 2001 to 2020. Proportion_loss denotes the proportion of mining-induced forest loss within PAs relative to the total mining-induced forest loss. Type A does not account for the PA establishment year and calculates mining-induced forest loss based on all PAs. Type B excludes PAs established after 2000 and calculates mining-induced forest loss. Type C incorporates the PA establishment year: for PAs established before 2000, mining-induced forest loss is calculated from 2001 to 2020; for PAs established after 2000, mining-induced forest loss is calculated from the PA establishment year to 2020. Source data are provided as a Source Data file.

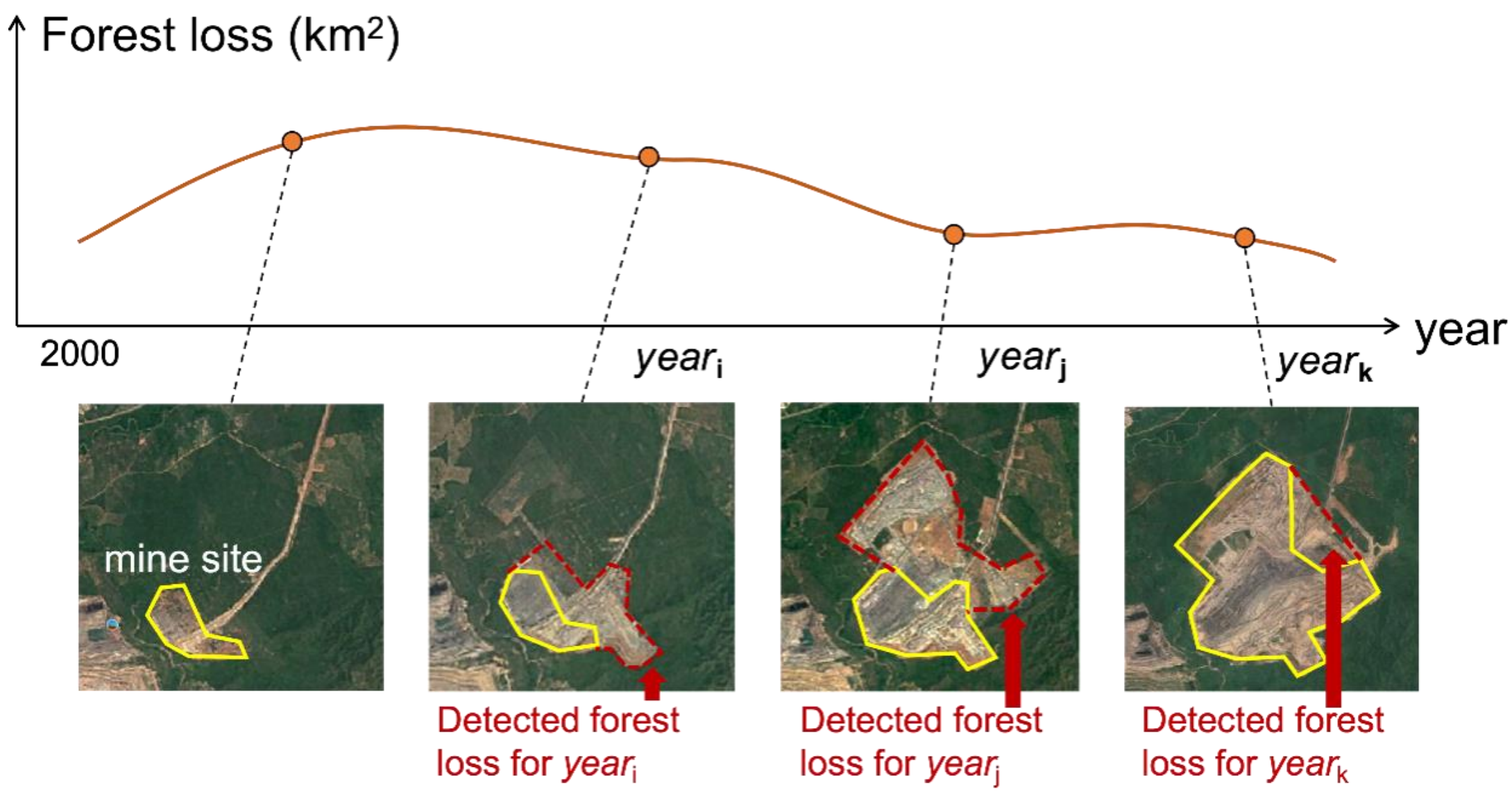

Supplementary Fig. 14. Conceptual diagram of active mine site classification

The figure illustrates a mine site expanding over the study period (from year *i* to year *k*) and the associated forest loss, based on Google Earth satellite imagery (Map data: Google Earth, © Maxar Technologies).

Supplementary Table 1. Post-mining forest gain statistics

AF (Africa), AS (Asia), OC (Oceania), EU (Europe), SA (South America), and NA (North America) represent different continents, and Global refers to the global scale. Recovery (%) is defined as the ratio of total forest gain to total forest loss. See Supplementary Note 1 for details.

Continent	Total forest gain (km^2)				Total forest loss (km^2)				Recovery (%)			
	Total	Within PAs	Beyond PAs (5 km buffer)	Beyond PAs (10 km buffer)	Total	Within PAs	Beyond PAs (5 km buffer)	Beyond PAs (10 km buffer)	Total	Within PAs	Beyond PAs (5 km buffer)	Beyond PAs (10 km buffer)
AF	21	0.31	9.97	16.6	282	31.9	122	185	7.45	0.97	8.17	8.97
AS	102	1.53	5.57	16	2397	39.2	181	418	4.26	3.90	3.08	3.83
OC	7.7	0.02	3.65	4.8	211	3.9	78	128	3.65	0.51	4.68	3.75
EU	0.7	0.04	0.28	0.5	161	6.0	97	126	0.43	0.67	0.29	0.40
SA	28.1	5.6	2.66	8.1	1140	222.9	173	391	2.46	2.51	1.54	2.07
NA	28.9	0.18	3.80	7.8	1519	9.6	208	472	1.90	1.88	1.83	1.65
Global	188.4	7.68	25.93	53.8	5710	313.5	859	1720	3.30	2.45	3.02	3.13

Supplementary Table 2. Active mine sites within protected areas (PAs) at the national scale

National-scale counts of total and active mine sites within PAs. Active mine sites exhibiting accelerating forest loss were identified using linear regression models based on annual forest loss during the study period.

Country	Total mine sites	Active mine sites	Active mine sites exhibiting accelerating forest loss
Australia	64	4	2
Bulgaria	14	2	2
Brazil	744	366	301
Canada	18	1	1
Chile	7	2	2
China	184	22	10
Côte d'Ivoire	8	5	5
DRC	12	2	2
Colombia	14	2	2
Cuba	4	2	1
Germany	59	5	2
Algeria	4	1	0
Spain	19	4	3
French Guiana	41	6	3
Ghana	73	30	26
Guinea	12	2	1
Greece	28	4	2
Guyana	4	3	2
Honduras	3	1	1
Hungary	23	1	1
Indonesia	46	6	6
India	5	1	0
Ireland	1	1	0
Italy	3	1	0
South Korea	5	1	1
Madagascar	2	1	1
Mexico	23	4	2
Myanmar	2	1	1
Mozambique	3	2	1
New Zealand	31	2	2
Philippines	38	2	1
Poland	17	3	3
Russia	56	1	1
Sierra Leone	18	4	2
Suriname	16	4	3

Slovakia	22	1	1
Tanzania	16	2	1
Ukraine	17	2	0
United States	75	2	2
Venezuela	90	33	30
Vietnam	11	3	2
Zambia	19	2	0

Supplementary Table 3. Forest loss within and beyond protected areas (PAs) at the national scale

National-scale forest loss within PAs and the corresponding forest loss beyond PAs (10-km buffer zones) during the study period. Ratio (%) denotes forest loss beyond PAs relative to that within PAs. Countries with cumulative forest loss within PAs below 0.001 km^2 were excluded.

Country	Forest loss within PAs (km^2)	Forest loss beyond PAs (km^2)	Ratio (%)
Albania	0.010	0.272	27.2
Armenia	0.005	1.888	377.6
Australia	4.571	241.188	52.8
Austria	0.181	0.453	2.5
Belgium	0.284	0.019	0.1
Bulgaria	2.462	3.074	1.2
Bolivia	1.726	4.952	2.9
Brazil	915.908	739.894	0.8
Canada	0.368	164.235	446.3
Chile	0.489	1.452	3.0
China	8.928	25.317	2.8
Côte d'Ivoire	3.632	23.233	6.4
DRC	3.316	12.622	3.8
Colombia	1.459	32.790	22.5
Cuba	0.168	11.920	71.0
Czechia	0.002	3.020	1510
Germany	5.159	15.602	3.0
Dominican Republic	0.114	9.404	82.5
Algeria	0.186	0.222	1.2
Ecuador	0.002	13.512	6756
Spain	0.181	8.637	47.7
Estonia	0.014	22.819	1629.9
French Guiana	9.781	35.563	3.6
United Kingdom	0.073	3.344	45.8
Ghana	23.554	500.839	21.3
Guinea	2.795	2.261	0.8
Greece	0.609	4.061	6.7
Guatemala	0.001	1.822	1822
Guyana	0.471	6.854	14.6
Honduras	0.088	0.675	7.7
Croatia	0.005	0.096	19.2
Hungary	0.269	0.523	1.9
Indonesia	37.900	572.395	15.1

India	0.006	0.111	18.5
Italy	0.001	0.489	489
Jamaica	0.015	7.332	488.8
Japan	0.031	1.854	59.8
Kazakhstan	0.003	0.020	6.7
Cambodia	0.110	1.389	12.6
South Korea	0.144	1.787	12.4
Lebanon	0.002	0.021	10.5
Madagascar	1.999	7.759	3.9
Mexico	3.862	7.085	1.8
Myanmar	12.642	117.264	9.3
Mozambique	0.328	0.994	3.0
Malawi	0.015	0.089	5.9
Malaysia	1.852	24.379	13.2
Nigeria	0.038	0.046	1.2
Nicaragua	0.237	1.289	5.4
Norway	0.001	1.154	1154
New Zealand	2.457	9.991	4.1
Panama	8.110	7.654	0.9
Peru	9.441	288.478	30.6
Philippines	7.227	22.572	3.1
Papua New Guinea	1.141	3.126	2.7
Poland	2.159	9.414	4.4
Portugal	0.028	1.937	69.2
Romania	0.026	3.942	151.6
Russia	8.916	206.236	23.1
Sierra Leone	2.625	10.681	4.1
El Salvador	0.004	0.030	7.5
Serbia	0.020	0.950	47.5
Suriname	11.531	100.955	8.8
Slovakia	0.244	0.737	3.0
Sweden	0.020	26.536	1326.8
Thailand	0.004	0.476	119
Tanzania	5.747	5.955	1.0
Uganda	0.006	0.032	5.3
Ukraine	0.083	8.727	105.1
United States	13.230	533.893	40.4
Venezuela	213.228	76.626	0.4
Vietnam	0.710	10.708	15.1
South Africa	0.020	7.041	352.1
Zambia	39.165	79.212	2.0
Zimbabwe	0.002	0.017	8.5

Supplementary Table 4. Total CO_2 emission associated with mining-induced forest loss from 2001–2020

AF (Africa), AS (Asia), OC (Oceania), EU (Europe), SA (South America), and NA (North America) represent different continents, and Global refers to the global scale. Carbon emissions were calculated as the sum of soil organic carbon (Soil), average aboveground biomass carbon (Aboveground), and average belowground biomass (Belowground). Data of Aboveground, Belowground, and Sum are presented as mean ± Standard Deviation (SD). See Supplementary Note 2 for details.

Scale	Class	AF	AS	OC	EU	NA	SA	Global
	Aboveground	21.5±1.1	156.7±8.2	5.2±0.3	3.8±0.2	37.8±2.0	155.3±8.2	380.4±20.0
	Belowground	3.2±0.2	22.2±1.2	0.9±0.04	0.8±0.04	6.1±0.3	20.7±1.1	53.9±2.8
Global	Soil	2.05	25.3	1.0	4.2	25.8	11.8	70.15
	Sum	26.7±1.3	204.2±9.4	7.1±0.32	8.8±0.24	69.7±2.3	187.8±9.3	504.4±22.8
	Aboveground	2.2±0.1	2.41±0.1	0.18±0.01	0.24±0.01	0.63±0.03	48.2±2.5	53.9±2.8
Within	Belowground	0.39±0.02	0.44±0.02	0.04±0.002	0.06±0.003	0.12±0.006	8±0.42	9.1±0.48
PAs	Soil	0.204	0.464	0.033	0.085	0.086	3.12	3.99
	Sum	2.8±0.13	3.3±0.15	0.25±0.01	0.32±0.01	0.76±0.04	57.3±2.95	66.0±3.31
Beyond	Aboveground	15.6±0.8	29.3±1.5	3.6±0.2	2.9±0.1	14.0±0.7	52.8±2.7	118.3±6.2
PAs	Belowground	3.27±0.17	5.88±0.31	0.78±0.04	0.71±0.04	3.14±0.16	9.90±0.52	23.7±1.25
(10 km	Soil	1.45	3.76	0.73	3.45	3.37	4.34	17.1
buffer)	Sum	20.3±0.99	38.9±1.85	5.1±0.23	7.1±0.19	20.5±0.90	67.0±3.30	159.1±7.47

Supplementary Table 5. Global distribution of forest cover, mine sites, and their overlap with protected areas (PAs)

Statistic metric	Area (km^2)
Global forest cover area	50,901,434
Global forest cover area within PAs	8,550,481
Global forest cover area within mine sites	43,262
Mine site area within PAs	6,359
Forest area in the overlap between PAs and mine sites	3,265
Mining-induced forest loss within PAs	1,372

Supplementary References

1. Hansen, M. *et al*. High-resolution global maps of 21st-century forest cover change. *Science* **342,** 850–853 (2013).

2. Harris, N. *et al*. Global maps of twenty-first century forest carbon fluxes. *Nat. Clim. Chang.* **11**, 234–240 (2021).

3. Saleem, I. *et al*. Biomass production, carbon stock and sequestration potential of prominent agroforestry systems in north-western Himalaya, India. *Front. For. Glob. Chang.* **6**, 1192382 (2023).

4. Wang, M. *et al*. Mapping accumulated carbon storage of global mangroves from 2000 to 2020 at a 1 km resolution. *Sci. Data* **12**, 552 (2025).

5. Mugabowindekwe, M. *et al*. Nation-wide mapping of tree-level aboveground carbon stocks in Rwanda. *Nat. Clim. Chang.* **13**, 91–97 (2023).

6. Feng, Y. *et al*. Doubling of annual forest carbon loss over the tropics during the early twenty-first century. *Nat. Sustain.* **5**, 444–451 (2022).

7. Brinck, K. *et al*. High resolution analysis of tropical forest fragmentation and its impact on the global carbon cycle. *Nat. Commun.* **8**, 14855 (2017).

8. Saatchi, S. *et al*. Benchmark map of forest carbon stocks in tropical regions across three continents. *Proc. Natl. Acad. Sci. U.S.A.* **108**, 9899–9904 (2011).

9. Koh, L. *et al*. Carbon prospecting in tropical forests for climate change mitigation. *Nat. Commun.* **12**, 1271 (2021)

10. Mokany, K. & Prokushkin, A. Critical analysis of root: shoot ratios in terrestrial biomes. *Glob. Chang. Biol.* **12**, 84–96 (2006).

11. Panagos, P. *et al*. European Soil Data Centre: Response to European policy support and public data requirements. *Land Use Policy* **29**, 329–338 (2012).

12. Don, A. *et al*. Impact of tropical land-use change on soil organic carbon stocks-a meta-analysis. *Glob. Chang. Biol.* **17**, 1658–1670 (2011).

13. Avitabile, V. *et al*. An integrated pan-tropical biomass map using multiple reference datasets. *Glob. Chang. Biol.* **22**, 1406–1420 (2016).

14. Santoro, M. & Cartus, O. ESA Biomass Climate Change Initiative (Biomass_cci): Global datasets of forest above-ground biomass for the years 2010, 2015, 2016,

2017, 2018, 2019, 2020 and 2021, v5. NERC EDS Centre for Environmental Data Analysis. https://dx.doi.org/10.5285/02e1b18071ad45a19b4d3e8adafa2817 (2024).

15. Spawn, S.A., Sullivan, C.C., Lark, T.J. & Gibbs, H.K. Harmonized global maps of above and belowground biomass carbon density in the year 2010. *Sci. Data* **7**, 112 (2020).
16. Maus, V. *et al*. An update on global mining land use. *Sci. Data* **9**, 433 (2022).
17. Tang, L. & Werner, T. T. Global mining footprint mapped from high-resolution satellite imagery. *Commun. Earth Environ.* **4**, 134 (2023).
