# Supplementary material for: Global mining has undermined forest conservation within and beyond protected areas
Source: Nat Commun. 2026 Jun 24;17:7954. doi: 10.1038/s41467-026-74859-3 (PMC13448669; doi:10.1038/s41467-026-74859-3)
Supplement: Supplementary file 2 [file 41467_2026_74859_MOESM2_ESM.pdf]

nature portfolio

Peer Review File

Global mining has undermined forest conservation within and beyond protected areas

Corresponding Author: Professor Tingting He

This manuscript has been previously reviewed at another journal. This document only contains information relating to versions considered at Nature Communications.

This file contains all reviewer reports in order by version, followed by all author rebuttals in order by version.

Version 0:

Reviewer comments:

Reviewer #4

(Remarks to the Author)
Overall assessment

I have now revised the letter to the reviewers prepared by the authors, as well as the manuscript with track changes and the supplementary materials provided. I find the results of this study are noteworthy and, though not completely novel, I think the study can be published in Nature Communications. However, in my view the authors must still make several minor corrections to further improve the clarity of the manuscript and the significance of their results, including the potential impact for conservation and minging policies. In what follows, I highlight several issues that deserve attention. They all should be fairly straightforward to address.

Main issues

– Put results in greater context: I miss having some results compared to the total amount or coverage worldwide. For instance, what is the global extent of forests? And of protected areas? And of mines? And of forests within PAs? And of forests and mines within PAs? Having this sort of figures in place would help the readers better understand the significance of the results.

– Clarify novelty and significance of results: it seems that the authors have improved these aspects (e.g., citing Luckeneder et al.'s paper), but I think there is still room for improvement here. For instance, whereas Luckeneder et al. they report a forest loss of 8% in PAs, the 11% reported here is probably significantly higher. Yet, this potential significance is not discussed and, I think to recall, not even mentioned in the text as such. Also, are not other similar studies aside from Luckeneder et al.? Novelty is not sufficiently addressed. In addition, the significance of results is neither stated nor is it discussed in terms of policy or other potential solutions in relation to mining-induced forest loss in and beyond PAs.

– Improve the discussion section: this is the main issue that must be improved in my opinion. This section is divided into three parts: 1) an expansion of results (LL 254–291); 2) a discussion of the main limitations of the study (LL 293–319); and 3) a brief conclusion (LL 321–338). Several things come to mind. To begin with, the first part should not be a part of a discussion section, but either of the results section or of an annex (i.e., supplementary material). In addition, the only proper discussion focuses just on the limitations of the study identified by the authors, i.e., there is nothing regarding the discussion of results compared to previous findings, which is a key omission; in line with this, their academic significance is not discussed nor their value for policy-making. Finally, the first part of the conclusion is too general and the recommendations are well known, thus lacking novelty (LL 321–329). In addition, the second part (LL 329–338) is likewise rather general and lacks novelty. The authors should focus on more specific claims and, ideally, provide examples of policies or actions that have worked at different scales, or specific suggestions on how to deal with mining within and beyond PAs and how to put them forward.

Minor issues

– Improve title: I do not think that “is threatening” is very precise as the forest loss within PAs quantified by the authors implies an actual and significant impact, not a threat. Therefore, a more accurate title could be something like that: “Global mining has undermined forest conservation within and beyond protected areas” (just an example of several possibilities).

– Enhance figures: there are several issues that could be easily improved in Figures for the sake of greater legibility and consistency across figures. For instance, Fig. 1a and Fig. 2a could have the same color coding; same with Fig. 1b vs Fig. 2e and Fig. 1c vs Fig. 2d. Fig. 2a, d, e: color coding is actually counter-intuitive. Fig 2c: why is forest loss in the X axis while elsewhere it is placed in the Y axis? Some extended figures could easily be clearer is the authors were consistent with the Y axis scale within each figure, e.g., 1, 3, 5, 7; this would allow for better comparison of results between regions. Fig. 6a, b, c: where are the three windows located in the bigger map? Does the scale bar refer to the big map or to the windows? Unclear.. Fig. 7: why do the color of each map changes between a and b? Confusing and unnecessary. Fig. 8: I do not understand this figure. Use a different color for Maus' as green coincides with the forest backdrop. The figure caption should be self-explanatory. Fig. 9: it is clear but it calls into question the methodological procedure to classify active vs inactive mines (see comment below).

– Missing tables: some tables are missing from the supplementary materials, which contains 9 additional figures.

– Methodological pitfalls: regarding the classification of active vs inactive mines, is there another way to verify what mines are actually inactive? It could also be that the mine hasn't expanded but deepened? Or simply that a mine that is not surrounded by forests do not lead to forest loss when it grows? (as you pointed out in your reply to Reviewer 1 in your response #7). What's the reliability of this classification?

– Edit and take care of several errors: the document would benefit from some edits to get rid of several errors including typos (e.g., the Borneo, the Congo: LL 123–124), incomplete sentences (e.g., L 271; ref. #24) or misplaced things (e.g., a call for comparison typical of discussion, but inappropriate for a results section: L. 127; calls for figures and tables in the discussion section: LL 258–288), among other issues.

Open Access This Peer Review File is licensed under a Creative Commons Attribution 4.0 International License, which permits use, sharing, adaptation, distribution and reproduction in any medium or format, as long as you give appropriate credit to the original author(s) and the source, provide a link to the Creative Commons license, and indicate if changes were made.
In cases where reviewers are anonymous, credit should be given to 'Anonymous Referee' and the source.
The images or other third party material in this Peer Review File are included in the article’s Creative Commons license, unless indicated otherwise in a credit line to the material. If material is not included in the article’s Creative Commons license and your intended use is not permitted by statutory regulation or exceeds the permitted use, you will need to obtain permission directly from the copyright holder.
To view a copy of this license, visit https://creativecommons.org/licenses/by/4.0/

Dear Referees,

Thanks for your careful review of our manuscript. We sincerely appreciate your valuable comments and suggestions, which have significantly helped us improved the quality of our manuscript. In response your valuable suggestions, we have made substantial revisions to the manuscript. We provide a point-by-point response (in **blue**) to each or your comments, and all corresponding changes in manuscript have been highlighted in **red**.

Reviewer #4 (Comments for the Author):

Comment 1. Overall assessments. I have now revised the letter to the reviewers prepared by the authors, as well as the manuscript with track changes and the supplementary materials provided. I find the results of this study are noteworthy and, though not completely novel, I think the study can be published in Nature Communications. However, in my view the authors must still make several minor corrections to further improve the clarity of the manuscript and the significance of their results, including the potential impact for conservation and mining policies. In what follows, I highlight several issues that deserve attention. They all should be fairly straightforward to address.

Response: We sincerely thank the reviewer for the positive and encouraging overall assessment of our manuscript. In response the reviewer's suggestion, we have carefully revised the manuscript to further improve the clarity of presentation and to better highlight the significance of our findings. We have strengthened the discussion of the broader implications of our results. We had addressed all specific comments in detail below.

Comment 2. Put results in greater context: I miss having some results compared to the total amount or coverage worldwide. For instance, what is the global extent of forests? And of protected areas? And of mines? And of forests within PAs? And of forests and mines within PAs? Having this sort of figures in place would help the readers better understand the significance of the results.

Response: We thank the reviewer for this helpful suggestion. In response, we have added a new table (Supplementary Table S5), which summarizes key global reference metrics. This additional context allows readers to better interpret the relative significance of our estimates.

Also, we revised the Discussion section (page 14, lines 238-243; 252-255) to explicitly compare mining-induced forest loss with relevant baseline extents in the revised manuscript, as follows:

Discussion (page 14, lines 238-243):

"Our results show that between 2000 and 2020, ~11% (~1,372 km^2) of global mining-induced forest loss occurs within PAs, while over 30% occurs within a 10-km buffer outside PA boundaries. Although this magnitude of loss may appear modest in a global context, it becomes substantial when considered against the forest area overlapping PAs and mine sites (~3,265 km^2), representing ~42% loss of forest within PAs over the study period (Supplementary Table S5)."

Discussion (page 14, lines 252-255):

"For example, some mine sites that spatially overlap with PAs do not necessarily result in forest loss. We find that the actual forest loss area within PAs is substantially smaller than the area of mine sites within these areas (Supplementary Table S5)."

Table S5. Global distribution of forest area, mine sites, and their overlap with PAs.

	Global forest area (km^2)	Global forest area within PAs (km^2)	Global forest area within mine sites (km^2)
Area	50901434	8550481	43262
	Mine site area within PAs (km2)	Forest area in the overlap between PAs and mine sites (km^2)	Mining-induced forest loss within PAs (km^2)
Area	6359	3265	1372

Comment 3. Clarify novelty and significance of results: it seems that the authors have improved these aspects (e.g., citing Luckeneder et al.'s paper), but I think there is still room for improvement here. For instance, whereas Luckeneder et al. they report a forest loss of 8% in PAs, the 11% reported here is probably significantly higher. Yet, this potential significance is not discussed and, I think to recall, not even mentioned in the text as such. Also, are not other similar studies aside from Luckeneder et al.? Novelty is not sufficiently addressed. In addition, the significance of results is neither stated nor is it discussed in terms of policy or other potential solutions in relation to mining-induced forest loss in and beyond PAs.

Response: We thank the reviewer for this insightful suggestion. We have revised the manuscript to further clarify the novelty and significance of our study. First, we have strengthened the Introduction by incorporating additional relevant studies, thereby better positioning our work within the existing literature and highlighting its novelty (page 3, lines 47-66), as follows:

Introduction (page 3, lines 47-66):

"Approximately 14% of PAs contain mine sites within or nearby their boundaries[16], and since 2000, nearly half of global mineral extraction has occurred within 20 km of PAs[17]. A recent study also indicates that more than 7,000 PAs worldwide have been or are currently affected by mining activities[28]. These findings

provide global evidence of the growing exposure of PAs to mining pressure. However, most existing assessments infer potential impacts based primarily on spatial overlap between mining activities and PAs, which does not capture the actual extent or dynamics of mining-induced forest loss. Although an increasing number of regional case studies have documented forest loss within and beyond PAs attributable to mining[29,30,31,32,33], these studies remain spatially limited and lack a consistent global assessment. This limitation is particularly critical for areas surrounding PAs, where mining pressures may be closely linked to those inside PAs boundaries and disproportionately affect adjacent habitats and ecosystems that are often more vulnerable than the PAs themselves[34,35]. As global demand for critical minerals continues to surge under the ongoing energy transition[36,37,38,39,40], these pressures are expected to intensify further, amplifying risks to forest ecosystems associated with PAs. Despite these growing concerns, a systematic and spatially explicit global quantification of mining-induced forest loss both within and surrounding PAs remains lacking. Addressing this gap is essential for accurately evaluating the effectiveness of PAs under increasing resource extraction pressure and for informing evidence-based conservation strategies."

Besides, we explicitly address the difference between our findings and previous estimates. We also have expanded the discussion of the broader significance of our findings, including their implications for conservation effectiveness and policy-making. In particular, we emphasize the need for improved management of mining within and beyond PAs and propose targeted policy recommendations and potential solutions (specific revision can be found in our response to **Comment 4**).

Comment 4. Improve the discussion section: this is the main issue that must be improved in my opinion. This section is divided into three parts: 1) an expansion of results (LL 254–291); 2) a discussion of the main limitations of the study (LL 293–319); and 3) a brief conclusion (LL 321–338). Several things come to mind. To begin with, the first part should not be a part of a discussion section, but either of the results section or of an annex (i.e., supplementary material). In addition, the only proper discussion focuses just on the limitations of the study identified by the authors, i.e., there is nothing regarding the discussion of results compared to previous findings, which is a key omission; in line with this, their academic significance is not discussed nor their value for policy-making. Finally, the first part of the conclusion is too general and the recommendations are well known, thus lacking novelty (LL 321–329). In addition, the second part (LL 329–338) is likewise rather general and lacks novelty. The authors should focus on more specific claims and, ideally, provide examples of policies or actions that have worked at different scales, or specific suggestions on how to deal with mining within and beyond PAs and how to put them forward.

Response: We thank the reviewer for this insightful suggestion. We have substantially revised the *Results* and *Discussion* sections to address your concerns.

Firstly, we agree that the initial part of the Discussion contained result-oriented descriptions. These have now been relocated to the Results section and Supplementary Materials (Page 7, Line 127-131; Page 11, Line 178-181, 191-198; Page 12, Line 208-213).

Results (page 7, lines 127-131):

"*Despite the increasing extent of forest loss, forest recovery within PAs remains extremely limited. Using forest gain information from GFC dataset for 2001-2012, we assess post-mining forest recovery and find that forest gain within PAs accounts for only 2.45% of the total forest loss during this period (Supplementary Table S1).*"

Results (page 11, lines 178-181):

"Similar to patterns observed within PAs, forest recovery outside PAs remains extremely limited. During 2001-2012, forest gain within the 5-km and 10-km buffer zones accounts for only 3.02% and 3.13% of the corresponding forest loss, respectively (Supplementary Table S1)."

Results (page 11, lines 191-198):

"*Although some mining activities comply with PA regulations (e.g., prohibitions on mining with strict nature reserves), mining-driven forest loss exhibits a pronounced spatial expansion along the edges of PAs (Extended data Fig. 4). This expansion may strengthen the spatial connectivity of forest disturbances inside and outside PAs, thereby increasing pressure on forest conservation within these areas. Moreover, mining surrounding different PAs sometimes form interconnected spatial patterns, which may further undermine the integrity of forest ecosystems at the regional scale.*"

Results (page 12, lines 208-213):

"*Given the higher carbon storage potential of these forests, this trend may exacerbate global carbon loss. We estimate that mining-induced forest loss during the study period results in ~504±22.8 Mt CO_2 emissions (Supplementary Table S4; Supplementary Fig. S4), of which ~13% (~66±3.3 Mt) occurs within PAs and ~30% (~159±7.5 Mt) within their 10-km buffer zones."*

Second, we have thoroughly restructured the *Discussion* section to explicitly compare our findings with previous global-scale studies and to highlight the academic significance of our work. This includes a new paragraph discussing consistencies and discrepancies with prior research (Page 13, Line 224-259), as follows:

Discussion (page 13, lines 224-259):

"*Driven by substantial economic benefits and the growing demand for renewable energy materials, mining has expanded rapidly and become a major driver of deforestation. A considerable proportion of mine sites spatially overlap with PAs[16,17], raising concerns about their effectiveness in conserving forest ecosystems. This study*

provides a global-scale quantitative assessment of mining-induced forest loss within and around PAs. Previous studies have primarily relied on spatial analysis of mining activities and PAs to infer potential impacts. For example, one study reports that ~7% of 1,418 mines associated with four key metals directly overlap with PAs, while ~27% are located within 10 km of PA boundaries[16]*. Another study, based on ore extraction data from ~3,000 mine sites between 2000 and 2019, indicates that 50% of extraction occurred within 20 km of PAs, with 8% (~480 Mt) occurring inside PAs*[17]*. More recently, a study estimates that over 7,000 PAs worldwide have been or are currently affected by mining activities*[28]*. By integrating global forest change data, mining land-use data, and PA boundary datasets, we further quantify the actual dynamics of forest loss attributable to mining. Our results show that between 2000 and 2020, ~11% (~1,372 km*^{2}*) of global mining-induced forest loss occurs within PAs, while over 30% occurs within a 10-km buffer outside PA boundaries. Although this magnitude of loss may appear modest in a global context, it becomes substantial when considered against the forest area overlapping PAs and mine sites (~3,265 km*^{2}*), representing ~42% loss of forest within PAs over the study period (Supplementary Table S5). Our study reveals the direct contribution of mining to deforestation and, while not strictly comparable, is broadly consistent with previous findings and reinforces evidences of the substantial threat posed by mining to PAs. Our estimates are more conservative than those reported in ref*[28]*, likely due to the use of a more comprehensive global mining inventory in that study (>230,000 mining areas). Given the substantial gaps in global mining datasets*[39]*, our estimates may underestimate the negative impacts of mining on PAs and even global forests. Moreover, although these studies help identify potential threats posed by mining to PAs, they may not accurately capture the actual deforestation impacts of mining activities. For example, some mine sites that spatially overlap with PAs do not necessarily result in forest loss. We find that the actual forest loss area within PAs is substantially smaller than the area of mine sites within these areas (Supplementary Table S5). Conversely, some mining activities cause limited forest loss within PA boundaries but may trigger extensive deforestation in surrounding areas (Supplementary Table S3). These findings further highlight that differences in data completeness and methodological frameworks can substantially influence assessments of mining threats to PA ecosystems.*"

Third, we have expanded the discussion on study limitations by incorporating additional methodological considerations, particularly regarding the conservative classification of active mine sites (<u>page 16, lines 286-296; specific revision can be found in our response to Comment 8</u>). Finally, based on the specific findings of this study, we proposed targeted policy recommendations and potential solutions for addressing mining activities within and around PAs (<u>page 16, lines 298-333</u>).

Discussion (page 16, lines 298-333):

"*PAs are being expanded globally to protect forests and biodiversity. However, our results reveal that forests within existing PA boundaries have already been*

substantially degraded by mining. Although annual mining-related forest loss shows a declining trend, deforestation within PAs continues to intensify. These findings underscore the need not only to enforce stricter regulations on mining, but also to strengthen periodic review and adaptive oversight of existing mining concessions within PAs. Protection strategies confined to PA boundaries are insufficient to curb mining-induced forest loss, as surrounding areas experience even greater deforestation. Conservation strategies should therefore recognize PAs as integral components of broader ecological networks rather than isolated units[52,53]. Given that mining impacts can extend over tens of kilometers[27], there is an urgent need to establish buffer zones around PAs[54,55], restore critical ecological corridors[56], and maintain the connectivity and integrity of PAs networks[57].

Conflicts between conservation and development represent a key constraint on the effectiveness of PAs in protecting forests. In resource-dependent developing countries, blanket bans on mining may face significant implementation challenges and restrict local communities access to land and natural resources. For example, in Brazil, at least 20% of strictly PAs are under consideration for mining development[15,58]. Downgrading or opening existing PAs may further exacerbate indirect deforestation and forest fragmentation[33]. Therefore, prioritizing the protection of critical areas, while reducing secondary human pressures induced by mining (e.g., settlement expansion, agricultural development; ref[26]) through land use alternatives and compensation schemes, may offer a viable pathway.

Our findings also highlight the importance of integrating mining-related forest carbon loss accounting and compensation mechanisms into conservation and development decision-making. Mining-induced deforestation increasingly affects forests with higher biomass density both within and beyond PAs, leading to rising carbon emissions per unit of forest loss. Accordingly, differentiated management strategies across PAs, together with updates to the PAs classification[59], are needed to prioritize high priority forests and restrict or prohibit new mining activities[60]. Complementary measures, including compensation mechanisms61 and post-mining forest restoration requirements[47], could further ensure that mining activities internalize their ecological and climate responsibilities throughout the entire development lifecycle, thereby mitigating long-term environmental impacts."

Comment 5. Improve title: I do not think that "is threatening" is very precise as the forest loss within PAs quantified by the authors implies an actual and significant impact, not a threat. Therefore, a more accurate title could be something like that: "Global mining has undermined forest conservation within and beyond protected areas" (just an example of several possibilities).

Response: We thank the reviewer for this insightful suggestion. In line with the reviewer's suggestion, we have revised the title to better reflect the demonstrated effects of mining on forest conservation, as "*Global mining has undermined forest conservation within and beyond protected areas*".

Comment 6. Enhance figures: there are several issues that could be easily improved in Figures for the sake of greater legibility and consistency across figures. For instance, Fig. 1a and Fig. 2a could have the same color coding; same with Fig. 1b vs Fig. 2e and Fig. 1c vs Fig. 2d. Fig. 2a, d, e: color coding is actually counter-intuitive. Fig 2c: why is forest loss in the X axis while elsewhere it is placed in the Y axis? Some extended figures could easily be clearer is the authors were consistent with the Y axis scale within each figure, e.g., 1, 3, 5, 7; this would allow for better comparison of results between regions. Fig. 6a, b, c: where are the three windows located in the bigger map? Does the scale bar refer to the big map or to the windows? Unclear. Fig. 7: why do the color of each map changes between a and b? Confusing and unnecessary. Fig. 8: I do not understand this figure. Use a different color for Maus' as green coincides with the forest backdrop. The figure caption should be self-explanatory. Fig. 9: it is clear but it calls into question the methodological procedure to classify active vs inactive mines (see comment below).

Response: We thank the reviewer for these helpful suggestions, which has significantly improved the clarity, consistency, and interpretability of figures. We have revised the figures throughout the manuscript accordingly. All revised figures and corresponding Extended data figures are provided in the updated manuscript.

Specifically:

- **Color schemes:** We have standard the color coding across related panels to improve consistency (In particular, **Fig. 1a** and **Fig. 2a** now use the same color scheme, as do **Fig. 1b** and **Fig. 2e**, and **Fig.1c** and **Fig. 2d**);
- **Axis consistency:** In Fig. 2c, forest loss has been moved to the y-axis to ensure consistency with other figures in the manuscript.
- **Consistency in Extended data Figures 1,3,5,6:** The y-axis scales in Extended data Figs. 1, 3, 5, and 6 have been standardized to facilitate clearer comparison across regions.
- **Clarification of scale bar (Extended data Fig. 4):** Extended data Fig. 4 has been substantially revised. We now explicitly indicate the locations of the three zoom-in windows within the larger map, and clarify that the scale bar refers to the corresponding map panels.
- **Consistency in color (Extended data Fig. 6):** The color schemes in Extended data Fig. 6a and 6b have been unified to ensure consistency when representing the same biomass dataset.
- **Improvement of figure clarity (Extended data Fig. 7):** Extended data Fig. 7 has been redesigned for better clarity. In particular, we have adjusted the color scheme to improve contrast with the forest background and revised the caption to ensure it is self-explanatory.
- **Clarification related to active mines classification:** We acknowledge the reviewer’s concern regarding the classification of active versus inactive mines. This issue is addressed in detail in our response to the Comment 8 and

the description has been revised accordingly in both the *Methods* (page 29, lines 587-598; page 30, lines 607-613) and *Discussion* sections (page 16, lines 286-296). Also, we have redrawn the Extended data Fig. 8 to show a clear presentation.

Comment 7. Missing tables: some tables are missing from the supplementary materials, which contains 9 additional figures.

Response: Thank you for your valuable comment. We have carefully checked the supplementary materials and ensured that all relevant content is now included. A 8structured table of contents has also been added for clarity. The Supplementary Material now contains Supplementary Text (S1, S2), Supplementary Figures (Figures S1-S6), and Supplementary Tables (Tables S1-S5).

Comment 8. Methodological pitfalls: regarding the classification of active vs inactive mines, is there another way to verify what mines are actually inactive? It could also be that the mine hasn't expanded but deepened? Or simply that a mine that is not surrounded by forests do not lead to forest loss when it grows? (as you pointed out in your reply to Reviewer 1 in your response #7). What's the reliability of this classification?

Response: We thank the reviewer for raising this important point. We agree that mining activity does not always lead to detectable forest loss. As the reviewer correctly pointed out, some mines may continue operating without causing additional forest loss, for example, due to i) underground expansion, ii) absence of surrounding forest, or iii) continued operations within previously cleared areas (e.g., pits). In such cases, our approach may classify these mines as inactive. However, we emphasize that the objective of our study is not identify all operational mines globally, but rather to detect those mines that are actively contributing to forest loss. Therefore, we define "active mines" based on the presence of recent or continuous mining-induced forest loss, which directly aligns with our research focus on forest conservation impacts. Importantly, this approach is relatively conservative. It may underestimate the number of operational mines, but it is unlikely to overestimate mining-induced forest loss or its impacts on forests within and around PAs. As a result, our classification prioritizes robustness in attributing forest loss to mining activities.

To improve transparency and methodological clarity, we first revised the conceptual figure (Extended data Fig. 8) to show a clear presentation. We also have revised the Methods section (page 29, lines 587-598; page 30, lines 607-613) to provide a more detailed description and explicitly acknowledged the limitations of this approach in the Discussion section (page 16, lines 286-296), as follows:

Methods (page 29, lines 587-598):

"*Identifying mine sites that continue to expand and drive forest loss is critical for understanding the persistent pressure of mining on forests, particularly for those that have already caused forest loss within PAs, where ongoing operations may further threaten forests both within and beyond their boundaries. We therefore first identify all mine sites associated with forest loss within PAs during the study period and subsequently assess their activity status. As the global mining dataset used in this study does not provide information on operational status or activity timelines, we develop an approach based on the temporal dynamics of forest loss to infer mining activity. Here, "active mine sites" are defined as those that exhibit continued expansion and detectable forest loss during the study period (Extended data Fig. 8). Specifically, mine sites are classified according to the temporal trajectories of mining-induced forest loss from 2000 to 2020 using the following criteria*:"

Methods (page 30, lines 607-613):

"Based on these criteria, mine sites are classified as either active or inactive. For each identified active mine, we further fit a linear regression model to its annual forest loss trajectory to assess whether the rate of forest loss is accelerating (slope>0). Notably, this approach focuses on mine sites associated with ongoing forest loss; as the classification of mine activity relies on detectable forest loss, it is unlikely to capture mine sites that remain operational but do not generate additional forest loss."

Discussion (page 16, lines 286-296):

"Moreover, we clarify that the classification of active mine sites in this study is based on detectable forest loss associated with mining expansion. Consequently, some operational mine sites that do not result in additional forest loss, such as those involving underground extraction, expansion within previously cleared areas (e.g., pits), or operations in non-forested surroundings, may be classified as inactive. However, our focus is on mine sites that have caused sustained forest loss within PAs in recent years. Also, mine sites that remain operational but do not generate additional forest loss are unlikely to exert substantial impacts on forest conservation within or around PAs during the study period. Therefore, our approach provides a conservative estimate of the number of active mine sites."

Comment 9. Edit and take care of several errors: the document would benefit from some edits to get rid of several errors including typos (e.g., the Borneo, the Congo: LL 123–124), incomplete sentences (e.g., L 271; ref. #24) or misplaced things (e.g., a call for comparison typical of discussion, but inappropriate for a results section: L. 127; calls for figures and tables in the discussion section: LL 258–288), among other issues.

Response: We thank the reviewer for your careful review and helpful suggestions. We have thoroughly revised the manuscript to address the issue.

Specifically, we have corrected typographical errors (e.g., Indonesian Borneo (Kalimantan), the Democratic Republic of the Congo (DRC)) and improve the overall clarity and readability of the text. In addition, we have carefully reorganized the manuscript to ensure a clear distinction between sections. Content inappropriate for the Results section has been moved to the Discussion, while misplaced references to figures and tables have been relocated to the appropriate sections.

Thank you for the in-principle acceptance of our manuscript. We are grateful to you and the reviewers for your time and effort throughout the evaluation process, which has substantially improved the quality of our work.

We have carefully addressed all items in the Checklist file and provided detailed point-by-point responses. The revised files have been updated to comply with Nature Communications journal's formatting, figure, supplementary information, data availability, and code availability requirements. In addition, we identified and corrected several minor numerical, grammar, and spelling errors during preparation of the final files. Importantly, these minor corrections do not affect the statistical significance of the results, the interpretation of the findings, or the overall conclusions of the study.

We hope that the revised materials meet the journal's requirements and look forward to the next stage of the publication process.

#Remaining comments from reviewers

No comments.

Response: We thank the reviewers for approving our revisions; these suggestions helped us improve the quality of our manuscript
