# Supplementary material for: Global mining has undermined forest conservation within and beyond protected areas
Source: Nat Commun. 2026 Jun 24;17:7954. doi: 10.1038/s41467-026-74859-3 (PMC13448669; doi:10.1038/s41467-026-74859-3)
Supplement: Supplementary file 3 [file 41467_2026_74859_MOESM3_ESM.pdf]

nature portfolio

Corresponding author(s): Tingting He

Last updated by author(s): Jun 6, 2026

Reporting Summary

Nature Portfolio wishes to improve the reproducibility of the work that we publish. This form provides structure for consistency and transparency in reporting. For further information on Nature Portfolio policies, see our Editorial Policies and the Editorial Policy Checklist.

Statistics

For all statistical analyses, confirm that the following items are present in the figure legend, table legend, main text, or Methods section.

n/a	Confirmed	
☐	☒	The exact sample size (*n*) for each experimental group/condition, given as a discrete number and unit of measurement
☒	☐	A statement on whether measurements were taken from distinct samples or whether the same sample was measured repeatedly
☐	☒	The statistical test(s) used AND whether they are one- or two-sided<br>*Only common tests should be described solely by name; describe more complex techniques in the Methods section.*
☒	☐	A description of all covariates tested
☒	☐	A description of any assumptions or corrections, such as tests of normality and adjustment for multiple comparisons
☐	☒	A full description of the statistical parameters including central tendency (e.g. means) or other basic estimates (e.g. regression coefficient) AND variation (e.g. standard deviation) or associated estimates of uncertainty (e.g. confidence intervals)
☐	☒	For null hypothesis testing, the test statistic (e.g. *F*, *t*, *r*) with confidence intervals, effect sizes, degrees of freedom and *P* value noted<br>*Give P values as exact values whenever suitable.*
☒	☐	For Bayesian analysis, information on the choice of priors and Markov chain Monte Carlo settings
☒	☐	For hierarchical and complex designs, identification of the appropriate level for tests and full reporting of outcomes
☒	☐	Estimates of effect sizes (e.g. Cohen's *d*, Pearson's *r*), indicating how they were calculated

Our web collection on statistics for biologists contains articles on many of the points above.

Software and code

Policy information about availability of computer code

Data collection	No software is used to collect data and all datasets are downloaded from sources using web browsers.
Data analysis	We used Google Earth Engine (Javascript) and ArcGIS 10.2 software to process spatial data.<br>We performed data analysis and visualization using R studio (version 1.3.1093). Related R packages are as follows: rnaturalearth(version 1.0.1), rnaturalearthdata (version 1.0.0), ggplot2 (version 3.5.1), trend (1.1.6), sf (version 1.0.7), dplyr (version 1.1.4), maps (version 3.4.2).<br>The data and codes required to reproduce the main figures are available at https://zenodo.org/records/20553030.

For manuscripts utilizing custom algorithms or software that are central to the research but not yet described in published literature, software must be made available to editors and reviewers. We strongly encourage code deposition in a community repository (e.g. GitHub). See the Nature Portfolio guidelines for submitting code & software for further information.

Data

Policy information about availability of data

All manuscripts must include a data availability statement. This statement should provide the following information, where applicable:

- Accession codes, unique identifiers, or web links for publicly available datasets
- A description of any restrictions on data availability
- For clinical datasets or third party data, please ensure that the statement adheres to our policy

Protected areas (PAs) are obtained from the February 2022 version of the World Database on Protected Areas (WDPA, https://www.protectedplanet.net/en/). The Hansen Global Forest Change (GFC, v1.10) dataset is available at https://storage.googleapis.com/earthenginepartners-hansen/GFC-2022-v1.10/download.html. The

Maus's (Version 2) and Tang's global mining land-use datasets are available at https://doi.pangaea.de/10.1594/PANGAEA.942325, and https://zenodo.org/records/6806817, respectively. The 2000 global forest management map is available at https://www.environmentalgeography.nl/site/data-models/data/forest-classes-and-uses/. The global aboveground live woody biomass density map is available at https://data.globalforestwatch.org/datasets/gfw::aboveground-live-woody-biomass-density/about. The GEOCARBON global aboveground forest biomass is available at https://doi.org/10.5281/zenodo.20534466. The Harmonized above/belowground biomass carbon density map can be accessed from https://developers.google.com/earth-engine/datasets/catalog/NASA_ORNL_biomass_carbon_density_v1?hl=zh-cn. The ESA CCI global above-ground biomass (v5.01) can be accessed from https://gee-community-catalog.org/projects/cci_agb/?h=esa+cci+agb.#dataset-preprocessing-for-gee. Source data are provided with this paper.

Research involving human participants, their data, or biological material

Policy information about studies with human participants or human data. See also policy information about sex, gender (identity/presentation), and sexual orientation and race, ethnicity and racism.

Reporting on sex and gender	NA
Reporting on race, ethnicity, or other socially relevant groupings	NA
Population characteristics	NA
Recruitment	NA
Ethics oversight	NA

Note that full information on the approval of the study protocol must also be provided in the manuscript.

Field-specific reporting

Please select the one below that is the best fit for your research. If you are not sure, read the appropriate sections before making your selection.

☐ Life sciences ☐ Behavioural & social sciences ☒ Ecological, evolutionary & environmental sciences

For a reference copy of the document with all sections, see nature.com/documents/nr-reporting-summary-flat.pdf

Ecological, evolutionary & environmental sciences study design

All studies must disclose on these points even when the disclosure is negative.

Study description	Using the Global Forest Change (GFC) dataset, global mining land-use datasets, and PA boundaries, we provided a global assessment of mining-induced forest loss within and beyond protected areas (PAs). We also integrated global biomass density map to examine temporal trends in biomass density associated with mining-induced forest loss.
Research sample	The study used global pixels for analysis, rather than specific samples. The data source has already been specified in the Data Availability section.
Sampling strategy	The study used global pixels for analysis, rather than specific samples.
Data collection	Protected areas (PAs) are obtained from the February 2022 version of the World Database on Protected Areas (WDPA, https://www.protectedplanet.net/en/). The Hansen Global Forest Change (GFC, v1.10) dataset is available at https://storage.googleapis.com/earthenginepartners-hansen/GFC-2022-v1.10/download.html. The Maus's (Version 2) and Tang's global mining land-use datasets are available at https://doi.pangaea.de/10.1594/PANGAEA.942325, and https://zenodo.org/records/6806817, respectively. The 2000 global forest management map is available at https://www.environmentalgeography.nl/site/data-models/data/forest-classes-and-uses/. The global aboveground live woody biomass density map is available at https://data.globalforestwatch.org/datasets/gfw::aboveground-live-woody-biomass-density/about. The GEOCARBON global aboveground forest biomass is available at https://doi.org/10.5281/zenodo.20534466. The Harmonized above/belowground biomass carbon density map can be accessed from https://developers.google.com/earth-engine/datasets/catalog/NASA_ORNL_biomass_carbon_density_v1?hl=zh-cn. The ESA CCI global above-ground biomass (v5.01) can be accessed from https://gee-community-catalog.org/projects/cci_agb/?h=esa+cci+agb.#dataset-preprocessing-for-gee. Source data are provided with this paper.
Timing and spatial scale	Mining-induced forest loss within and beyond PAs (2001-2020; 30-m); Temporal trends in biomass density associated with mining-induced forest loss (2001-2020; 30-m)
Data exclusions	Following WPDA guidelines and similar global studies, we considered only polygon layers, excluding point layers. We excluded protected areas (PAs) without the status "Designated", "Established", or "Inscribed", as well as those designated as UNESCO Man and Biosphere Reserves (MAB). We excluded PAs smaller than 900 m2 to ensure compatibility with the 30-m resolution GFC dataset. We remove the plantations of treecover2000 layer (GFC dataset) using global forest management classification map.
Reproducibility	Data processing and analysis steps were described in detail in the Methods section to ensure reproducibility.
Randomization	Randomization was not involved in this study. We only focused on mining-induced forest loss. For protected areas (PAs) grouped by different categories, it is based on the International Union for Conservation of Nature (IUCN) management category.

Blinding	Blinding was not relevant to this study. All analyses were based on publicly available spatial dataset (remote sensing datasets) and automated computational procedures, without subject involvement during data acquisition or analysis.

Did the study involve field work? ☐ Yes ☒ No

Reporting for specific materials, systems and methods

We require information from authors about some types of materials, experimental systems and methods used in many studies. Here, indicate whether each material, system or method listed is relevant to your study. If you are not sure if a list item applies to your research, read the appropriate section before selecting a response.

Materials & experimental systems

n/a	Involved in the study
☒	☐ Antibodies
☒	☐ Eukaryotic cell lines
☒	☐ Palaeontology and archaeology
☒	☐ Animals and other organisms
☒	☐ Clinical data
☒	☐ Dual use research of concern
☒	☐ Plants

Methods

n/a	Involved in the study
☒	☐ ChIP-seq
☒	☐ Flow cytometry
☒	☐ MRI-based neuroimaging

Plants

Seed stocks	NA
Novel plant genotypes	NA
Authentication	NA
